# Genetic ME–a visualization application for merging and editing pedigrees for genetic studies

**DOI:** 10.1186/s13104-015-1131-y

**Published:** 2015-06-16

**Authors:** Diem K. Bui, Yingda Jiang, Xin Wei, Maria C. Ortube, Daniel E. Weeks, Yvette P. Conley, Michael B. Gorin

**Affiliations:** Department of Ophthalmology, David Geffen School of Medicine–UCLA, Los Angeles, CA 90095 USA; Department of Biostatistics, Graduate School of Public Health, University of Pittsburgh, Pittsburgh, PA 15261 USA; Department of Human Genetics, Graduate School of Public Health, University of Pittsburgh, Pittsburgh, PA 15261 USA; Department of Health Promotion and Development, School of Nursing, University of Pittsburgh, Pittsburgh, PA 15261 USA; Department of Human Genetics, David Geffen School of Medicine–UCLA, Los Angeles, CA 90095 USA

**Keywords:** Algorithms, Human, Java, Pedigree, Software

## Abstract

**Background:**

In order to study the genetics of diseases more accurately and effectively, one often collects large families. Different members of a large family may provide differing information about the structure and make-up of their pedigree. Thus, software is needed to facilitate reconciliation of pedigrees collected independently from multiple informants from a single large family to create a unified pedigree that is based on the best composite information available.

**Findings:**

Our implementation demonstrates that Genetic ME performs merging in terms of adding, replacing and combining information from two pedigrees. Through a tracking process, all of the changes made to the data set for the individuals can be traced back to their original source material. A new pedigree structure can be easily visualized while reconciling disparate information from multiple pedigrees.

**Methods:**

We developed the Genetic Merging & Editing (Genetic ME) program, an open source Java application built on top of CraneFoot and Ghostscript, to support comparing, editing and merging of pedigrees collected from multiple sources in a visually-oriented manner.

**Conclusions:**

Genetic ME constitutes an ideal addition to software packages for reconciling pedigree information from multiple sources. Genetic ME provides a friendly graphical user interface, traces the changes made by users, and produces viewable merged pedigree structures able to be further used by other popular analysis programs.

**Electronic supplementary material:**

The online version of this article (doi:10.1186/s13104-015-1131-y) contains supplementary material, which is available to authorized users.

## Findings

### Background

Family-based genetic studies may entail working with large and complex pedigree files that contain multiple descent trees, multiple spouses, consanguinity or inbreeding. These extended pedigrees may be constructed by merging separately collected sub-pedigrees obtained from multiple different family members. Often the information obtained from different family members may be incomplete, inexact, or may be conflicting, particularly with respect to the identification of more distant family members and their clinical information. We have found that attempting to reconcile these disparate sub-pedigrees without interactive visual feedback is both laborious and error-prone. Although current programs such as PedMerge [1] offer some support for merging pedigrees, we wanted to be able to also support pruning ambiguous or less certain elements from one or more pedigrees. While it is straightforward to draw a given pedigree structure–for example, the CraneFoot [2] program provides excellent facilities for drawing pedigrees–CraneFoot only processes a single pedigree file at a time. Our Genetic Merging & Editing (Genetic ME) program was developed to interactively support comparing, editing, and merging two pedigrees, while providing visualizations of the original and merged pedigree structures as drawn in the background by CraneFoot. To the best of our knowledge, there is no visually-oriented application that allows for reconciling inconsistent familial information from multiple sources into a unified pedigree while tracking the modifications.

### Implementation

Built on top of CraneFoot [2] and Ghostscript, the Java program Genetic ME provides a graphical user interface (GUI) that allows users to view two pedigrees and their corresponding familial, demographic, clinical, and genotyping data side by side for easy comparison. Using the GUI, users can edit current individuals’ information, add new individuals, compare an individual’s data as indicated by each pedigree, and reconcile discordances using the reconciling attributes feature. Users can also generate a unified pedigree that is the best composite of the two displayed pedigrees by applying one of three merge algorithms: (1) Replacing an individual and his/her sublineage from one pedigree with the other, (2) Combining data from one pedigree into the other, (3) Appending individuals and sublineages from one pedigree to the other. Genetic ME will then display the unified pedigree with the new changes for user approval before any modifications are saved into a new composite file. All text files are stored in tab-delimited format and all graphic files in Portable Network Graphics (PNG) format. Tracking is implemented so that all changes made can be traced back to their original sources.

#### Input files

Genetic ME requires a standardized input format consisting of a configuration file, a pedigree file, and an optional metadata file. The configuration file is a text file specifying the options controlling the appearances of the pedigrees. The pedigree file is a tab-delimited text file containing a set number of fields (*e.g.*, name, father, and mother) in a pre-defined order. Each row in the pedigree file encodes an individual, and contains his/her identifying information, disease status, phenotypic and genotypic information that has been obtained either by verbal history or other sources. The metadata file, if available, is generated by previous runs of Genetic ME. Each row in the metadata file corresponds to a row in the pedigree file, and encodes tracking information for the individual of interest. Tracking is implemented at the field level so that any changes made to an individual can be recorded. If no metadata file is specified, then the application will create a default one with the name of the pedigree file as the data source.

#### Output files

The unified pedigree is saved as a new dataset consisting of a configuration file, a pedigree file, and a metadata file. Genetic ME is non-destructive to the source pedigree information. Users can also edit the source pedigree information prior to creating the unified pedigree. In this case, Genetic ME modifies the existing pedigree file and the existing or default metadata file. Whenever a pedigree is drawn, low, medium, and high-resolution graphic files in PNG format are created and stored. All text files and graphic files are accessible outside of the application.

#### Algorithm

**Fig. 1:**
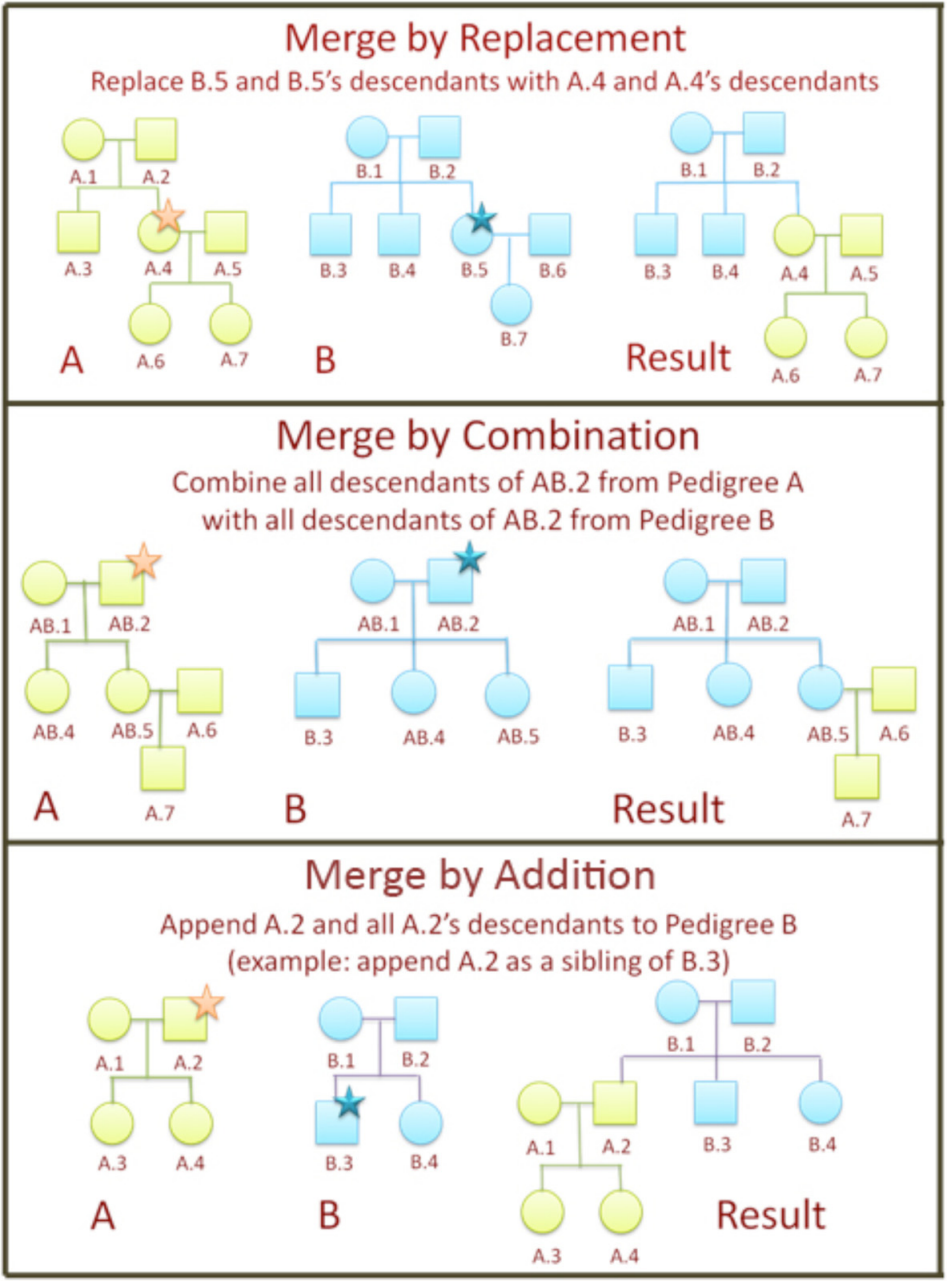
Examples illustrating merge by replacement, merge by combination, and merge by addition

To perform a merge action, the user specifies the direction of the merge (from source to destination), and the highest individual from each pedigree whose descendants are to be merged. The sublineage defined by the individual and his/her descendants forms the scope of operation for the algorithm. In all operations, there is no limit to the number of generations a sublineage can contain. All descendants will be merged. If a user desires to replace an individual without modifying his/her descendants, it is best to use the options for adding a new individual or removing an existing individual instead of the merge algorithms. An example of each of the merging algorithms is given below (Fig. 1). Note that during a merge operation, any duplicated individuals will be represented by only one person in the resulting merged pedigree. If these duplicated individuals have discordant phenotypes or genotypes, the algorithm will retain those from the destination pedigree, and discard those from the source pedigree. Hence, it is recommended that the user review and reconcile the discordances using the reconciling attributes feature prior to performing the merge operation.

#### Merge by replacing an individual or branch

In a “Merge by Replacement” operation, the user first chooses the direction of the merge (*e.g.*, from A to B, or from B to A), and then selects the individuals to involved. Figure 1 (top) illustrates what happens if the user chooses to merge A into B, replacing B.5 and B.5′s descendants with A.4 and A.4′s descendants. The resulting pedigree is the combination of pedigree B and the branch starting with person A.4 replacing the branch starting with person B.5.

#### Merge by combining data

In a “Merge by Combination” operation, the user chooses the merge direction and the heads of the sublineages to be combined in both pedigrees. Figure 1 (middle) illustrates what happens if the user chooses to combine all descendants of AB.2 from pedigree A with all descendants of AB.2 from pedigree B. The algorithm will combine all descendants of person AB.2 from pedigree A with all descendants of person AB.2 from pedigree B, and eliminate any duplicated individuals, retaining the attributes in the destination pedigree (as mentioned above). Additional iterations of merging by combination can be performed on the newly created unified pedigree and previous iteration’s source pedigree to create the best composite of the two original pedigrees.

#### Merge by adding individuals and sublineages

In a “Merge by Addition” operation, the user chooses the merge direction and the head of the sublineage that will be added to the target pedigree. Figure 1 (bottom) illustrates what happens if a user chooses to append A.2 and A.2′s descendants to pedigree B with A.2 as a sibling of B.3. The resulting merged pedigree contains pedigree B and the branch starting with person A.2 as a new sibling of person B.3.

### Discussion

Genetic ME is an ideal addition to software packages that facilitate the creation and verification of pedigrees [3] or integrate phenotypic and genotypic information [4] from large databases of genealogy data. A pedigree generated by any of those packages can be further modified by applications such as Genetic ME, PedMerge [1], or Pedigree Visualizer [5].

Although both Pedigree Visualizer [5] and PedMerge [1] are capable of invoking a merge or performing complex functions, Genetic ME has it own advantages in several aspects. Firstly, it is more convenient to enter individuals’ information in Genetic ME, which provides a user-friendly interface for data-entry procedures. Pedigree information stored in a text file can be directly input into Genetic ME for further display and manipulation. By contrast, Pedigree Visualizer calls for an input pedigree structure in Kleisli Exchange Format [5], which is not a commonly used format. PedMerge requires LINKAGE format files defining the pedigree structure that may not allow inclusion of individual’s name information [1]. Secondly, Genetic ME provides more advanced user interfaces. Neither Pedigree Visualizer nor PedMerge has user-friendly interfaces for data editing procedures–for those programs, pedigree information can only be modified in the input file. Genetic ME generates interactive dialog windows during data editing process. Thirdly, when manipulating pedigrees from different sources, Genetic ME can juxtapose two window frames for users to check changes in a direct comparison. This side-by-side display is not featured by Pedigree Visualizer or PedMerge. Fourthly, in addition to its graphical user interface, Genetic ME provides tracking mechanism for merge functions, and provides the ability to compare an individual’s data as indicated by each pedigree and reconcile the discordances.

While useful in its present form, GeneticME could be improved and extended in a number of ways. For example, it would be nice to implement the ability to ‘undo’ recent merge or editing actions and to zoom pedigree images, as well as to provide a facility for highlighting discrepancies between two pedigrees. GeneticME could also be extended to support exporting the final pedigree structures in more different formats. Such changes could be implemented by others, as the source code of GeneticME is included in the distribution (see Additional files 1 and 2).

Genetic ME is written in Java. It leverages the technology of the CraneFoot software package [2], a powerful and flexible open source pedigree drawing tool. It also requires Ghostscript to visualize the graphic files. It is supported under Windows, Macintosh and Linux operating systems.

## Availability and requirements

The GeneticME program, example data, and source code are freely available for download at https://sites.google.com/site/geneticmeapp, implemented in Java and supported on Windows, Macintosh, and Linux. It relies on two external C programs: CraneFoot and Ghostscript.

- **Project nam**e: Genetic ME (Genetic Merging & Editing)
- **Project home page:**https://sites.google.com/site/geneticmeapp
- **Operating system(s):** Windows 7, Mac OSX, and Debian Linux x86
- **Programming language:** Java
- **Other requirements:** Java 1.6 or higher, CraneFoot 3.2, and Ghostscript 9.00 or higher
- **License:** GNU General Public License (Version 3)
- **Any restrictions to use by non-academics:** none.
- **Contact:** Michael B. Gorin, MD, PhD (gorin@jsei.ucla.edu)

## Additional files

Additional file 1:
**Version 1.10 of the GeneticME package.**
Additional file 2:
**Version 1.10 of the GeneticME User Guide.**

## Abbreviations

Genetic ME: Genetic merging & editing
GUI: Graphical user interface
PNG: Portable network graphics
