# Supplementary material for: Genetic ME–a visualization application for merging and editing pedigrees for genetic studies
Source: BMC Res Notes. 2015 Jun 16;8:241. doi: 10.1186/s13104-015-1131-y (PMC4478623; doi:10.1186/s13104-015-1131-y)
Supplement: Additional file 1: — Version 1.10 of the GeneticME package. [file 13104_2015_1131_MOESM1_ESM.zip › GeneticME_v1.10/doc/Genetic_ME_User_Guide_v1.10.pdf]

Genetic ME – User Guide

A visualization application for merging and editing pedigrees for genetic studies

Version 1.1

Diem K. Bui, MD; Yingda Jiang, MB; Xin Wei, PhD; Maria C. Ortube, MD; Daniel E. Weeks, PhD; Yvette Conley, PhD; Michael B. Gorin, MD, PhD

10/16/2014

Table of Contents

1 Introduction 3
2 Installation 3
2.1 Windows Installation 4
2.2 Macintosh Installation 4
2.3 Linux Installation 6
3 Input/output 6
3.1 Input Files 6
3.1.1 Configuration File 7
3.1.2 Pedigree File 9
3.1.3 Metadata File 11
3.2 Output Files 11
3.2.1 Postscript (*.ps) File 11
3.2.2 Portable Network Graphics (*.png) File 11
3.2.3 Metadata (*.txt) File For Source Pedigree 11
3.2.4 Merged Dataset 11
4 Basic uses 12
4.1 Starting Genetic ME 12
4.2 Opening Pedigree Files 12
4.3 Editing Pedigree Data 14
4.3.1 By manually Changing the Data in the Table 14
4.3.2 By Adding a New Person 15
4.3.3 By Reconciling Attributes 18
4.4 Merging Pedigrees 19
4.4.1 By Combining Data from One Pedigree into Another 19
4.4.2 By Replacing an Individual or Branch from One Pedigree by the Other 21
4.4.3 By Adding Individuals and Sublineages from One Pedigree to the Other 24
5 References 26
6 Appendix 26

6.1 Example Configuration File 26
6.2 Example Pedigree File 28
6.3 Example Metadata File (as generated by Genetic ME) 29
7 License Agreement 30

1 INTRODUCTION

Genetic ME (Genetic Merging & Editing) is an open source Java application developed for the ease of viewing, editing and merging pedigree structures, and for comparing and reconciling incongruous clinical or genetic information of individuals collected from two or more different sources.

Genetic ME provides a graphical user interface (GUI) that allows users to view two pedigrees and their corresponding familial, demographic, clinical, and genotyping data side by side for easy comparison. The GUI eases the following tasks: (1) editing current individuals' information by manual modification, (2) comparing an individual's information as listed in pedigree A and pedigree B and reconcile any differences, and (3) adding new individuals or remove existing individuals.

Users can also reconcile differences between the two displayed pedigrees by applying one of the three merging algorithms: (1) Replacement of an individual or branch from one pedigree by the other, (2) Combination of data from one pedigree into another, and (3) Appending individuals and sublineages from one pedigree to the other. Individuals and data can be merged from pedigree A to pedigree B or vice versa. The application then displays the merged pedigree with the new changes for user approval before any modifications are saved into a new composite file. The original and resulting text files are stored in tab-delimited format and the graphic files in Portable Network Graphics (PNG) format. Tracking is implemented so that all changes made can be traced back to their original sources.

Genetic ME is written in Java 6 and runs on JRE 1.6. It leverages the technology of CraneFoot 3.2 (Mäkinen *et al.*, 2005), a powerful and flexible pedigree drawing tool. It also uses Ghostscript 9.00 to visualize the graphic files. It is supported in Windows, Macintosh OS X and Linux.

2 INSTALLATION

The Java application Genetic ME uses Java 1.6 or higher and requires CraneFoot and Ghostscript to be preinstalled. Compiled versions of CraneFoot 3.2 and Ghostscript 9.00 were used to test the application and implement all of the manipulations. These versions were built for and

tested with Windows 7, Intel-based Macintosh and Debian Linux x86. You may download the source code for CraneFoot and Ghostscript at:

- CraneFoot: http://www.finndiane.fi/software/cranefoot/
- Ghostscript: http://www.gnu.org/software/ghostscript/

Download the Genetic ME install package at:

- Genetic ME: https://sites.google.com/site/geneticmeapp/downloads/

2.1 Windows Installation

1. Unzip the Genetic ME installation package to C:\
2. It is required that the executable file GeneticME.jar is installed in "C:\GeneticME". It is also important to verify that the following files:
   - cranefoot.exe
   - gsdll32.dll
   - gsdll32.lib
   - gswin32.exe
   - gswin32c.exe

   are in "C:\GeneticME\bin". Note that the application depends on these files being installed to the correct locations. If either CraneFoot or Ghostscript is missing, or not installed in "C:\GeneticME", Genetic ME will fail to run properly.

3. Run the program by double-clicking the icon "C:\GeneticME\GeneticME.jar", or opening a command prompt window, going to "C:\GeneticME", and typing "GeneticME.jar".

2.2 Macintosh Installation

The Macintosh version of Genetic ME requires that 'cranefoot' and 'gs' executables be installed in your path. We provide two alternative approaches, A and B, for doing this. Where possible, we recommend pathway A, as we do not guarantee that our pre-compiled executables will be kept up to date or that they will work on your particular system.

1. Install 'cranefoot' and 'gs' executable programs in a folder that is in your path

A) Download the 'cranefoot' source code from http://www.finndiane.fi/software/cranefoot/ and compile it to an executable by opening a Terminal window, going to the 'source' directory in the cranefoot folder, and typing

```
g++ -O5 -o cranefoot main.cc *.cpp -lm
```

to invoke the compiler. When it finishes, you should have a new executable file 'cranefoot' in the directory. Move this into a directory that is in your path, such as /usr/local/bin. Note that this pathway requires that you first install the Xcode command-line compilation tools, available for free in the App store (On Mavericks OS X 10.9, these can be installed by typing "xcode-select –install" in the Terminal window). Similarly, install ghostscript – the easiest way to do this would be to use Richard Koch's installer available at his http://pages.uoregon.edu/koch/ website.

OR

B) Download the 'cranefoot' pre-compiled executable from the Genetic ME website, and place it in a directory that is in your path, such as /usr/local/bin. Install ghostscript in your path using Richard Koch's installer available at his http://pages.uoregon.edu/koch/ website.

2. Adjust your path so that Genetic ME knows where to find 'cranefoot' and 'gs'

Genetic ME is a graphical program, so one needs to adjust the path settings on your Macintosh so that it knows where to find 'cranefoot' and 'gs'. To do this:

2.1) Open a Terminal window

2.2) Type "sudo pico /etc/launchd.conf"

2.3) Add a line like this to this file:

```
setenv PATH /usr/bin:/bin:/usr/sbin:/sbin:/usr/local/bin
```

In this line above, we are adding the folder '/usr/local/bin' to the end of the default path, thus enabling Genetic ME to find and run 'cranefoot' and 'gs' if they are in that folder.

2.4) After saving the file, restart your computer.

3. Open a new Terminal window, and verify that 'cranefoot' and 'gs' are in your path by typing "which cranefoot" and "which gs". If these are in your path, then neither of these should respond with the phrase "command not found".
4. Unzip the Genetic ME installation package to your preferred directory.
5. Run the program either by double-clicking the icon “GeneticME.jar” or by typing “java -jar GeneticME.jar” in the Terminal window.

2.3 LINUX INSTALLATION

The Linux version of Genetic ME requires that 'cranefoot' and 'gs' executables be installed in your path. We provide two alternative approaches, A and B, for doing this. Where possible, we recommend pathway A, as we do not guarantee that our pre-compiled executables will be kept up to date or that they will work on your particular system.

1. Install 'cranefoot' and 'gs' executable programs in a folder that is in your path

A) Download the 'cranefoot' source code from http://www.finndiane.fi/software/cranefoot/ and compile it to an executable by opening a Terminal window, going to the 'source' directory in the cranefoot folder, and typing

```
g++ -O5 -o cranefoot main.cc *.cpp -lm
```

to invoke the compiler. When it finishes, you should have a new executable file 'cranefoot' in the directory. Move this into a directory that is in your path, such as /usr/local/bin. Similarly, install ghostscript. The easiest way would be to install Ghostscript from your Linux repository.

OR

B) Download the 'cranefoot' pre-compiled executable from the Genetic ME website, and place it in a directory that is in your path, such as /usr/local/bin. Install Ghostscript from your Linux repository.

2. Adjust your path so that Genetic ME knows where to find 'cranefoot' and 'gs'
3. Open a new terminal window, and verify that 'cranefoot' and 'gs' are in your path by typing "which cranefoot" and "which gs". If these are in your path, then neither of these should respond with the phrase "command not found".
4. Unzip the Genetic ME installation package to your preferred directory.
5. Run the program either by double-clicking the icon "GeneticME.jar" or by typing "java -jar GeneticME.jar" in the Terminal window.

3 INPUT/OUTPUT

3.1 INPUT FILES

Genetic ME requires a standardized input format consisting of a configuration file, a pedigree file, and an optional metadata file.

Genetic ME relies on CraneFoot to draw all pedigree diagrams. Hence, the specification requirements for the configuration file and pedigree file are mainly imposed by CraneFoot with additional restrictions required by Genetic ME. Details regarding these are outlined in the appropriate section below.

For additional information about CraneFoot, please refer to the published “CraneFoot v3.2 user’s guide” at http://www.finndiane.fi/software/cranefoot/.

3.1.1 Configuration File

1. This is a text file that specifies options to control the appearances of the pedigree diagram. The name of this file can be anything as long as it is in the format *<config-file-name>.txt*, for example “configA.txt”. Genetic ME will look for the pedigree file and an optional metadata file in the same location as the configuration file. If necessary, it will save the metadata file there as well. The configuration file must be readable by Genetic ME.
2. The configuration file contains the following file parameters:
   - PedigreeName (required):
     - CraneFoot does not impose any restriction regarding the syntax of this name. However, in order for Genetic ME to work, this must be specified as *<config-file-name>_results* in the configuration file. For example, in the “configA.txt” file, the PedigreeName must be specified as “configA_results”.
     - For each given PedigreeName, CraneFoot generates an associated **.eps* file for families, a **.ps* file for pedigree document, and a **.topology.txt file* for topology. However, Genetic ME only uses the **.ps* file for further processing and removes the **.eps* and **.topology.txt* files to avoid confusion. If you are interested in obtaining these two files, you can run CraneFoot as stand-alone application independent from Genetic ME.
   - PedigreeFile (required):
     - CraneFoot does not impose any restriction regarding the syntax of this name. However, in order for Genetic ME to work, this must be specified as *<config-file-name>_pedigree.txt* in the configuration file. For example, in the “configA.txt” file, the PedigreeFile must be specified as “configA_pedigree.txt”.
3. The configuration file contains the following structural parameters:
   - AgeVariable: Optional. Specified by the *SIBORD* column in the pedigree file. If used, need to be specified as *<config-file-name>_pedigree.txt* in the configuration file. This determines the order of siblings in the pedigree (oldest → youngest = left → right).

- FatherVariable: Required. Specified by the *FATHER* column in the pedigree file. Must be specified as *<config-file-name>_pedigree.txt* in the configuration file. This identifies the father individual in a pedigree graph.
- GenderVariable: Optional. Specified by the *GENDER* column in the pedigree file. If used, need to be specified as *<config-file-name>_pedigree.txt* in the configuration file. This determines the shape representing the individual (square or circle).
- MotherVariable: Required. Specified by the *MOTHER* column in the pedigree file. Must be specified as *<config-file-name>_pedigree.txt* in the configuration file. This identifies the mother node in a pedigree graph.
- NameVariable: Required. Specified by the *NAME* column in the pedigree file. Must be specified as *<config-file-name>_pedigree.txt* in the configuration file. This must be unique for each individual for identification.
- SubgraphVariable: Optional. Specified by the *FAMILY* column in the pedigree file. If used, need to be specified as *<config-file-name>_pedigree.txt* in the configuration file. This identifies the family.

4. The configuration file contains the following visualization parameters:
   - ArrowVariable: Optional. Specified by the *INDEX* column in the pedigree file. If used, need to be specified as *<config-file-name>_pedigree.txt* in the configuration file. Description from CraneFoot v3.2 user's guide: black arrowhead pointing to the node, drawn if integer value greater than 0.
   - ColorVariable: Optional. Specified by the *RISK* column in the pedigree file. If used, need to be specified as *<config-file-name>_pedigree.txt* in the configuration file. Description from CraneFoot v3.2 user's guide: individual background color. Valid codes within [000000, 999999].
   - PatternVariable: Optional. Specified by the *DISEASE* column in the pedigree file. If used, need to be specified as *<config-file-name>_pedigree.txt* in the configuration file. Description from CraneFoot v3.2 user's guide: individual patterns. Valid codes within [1, 99].
   - SlashVariable: Optional. Specified by the *DEAD* column in the pedigree file. If used, need to be specified as *<config-file-name>_pedigree.txt* in the configuration file. Description from CraneFoot v3.2 user's guide: diagonal slash, drawn if integer value greater than 0.
   - ShapeVariable: Optional. Specified by the *OCCUPATION* column in the pedigree file. If used, need to be specified as *<config-file-name>_pedigree.txt* in the configuration file. Description from CraneFoot v3.2 user's guide: shapes of pedigree nodes. Valid codes within [1, 8]. Cannot be used simultaneously with GenderVariable.
   - TextVariable (multiple): Optional. Specified by *NAME*, *GTYPE_A* and *GTYPE_B* columns in the pedigree file. If used, need to be specified as *<config-file-name>*-

_pedigree.txt in the configuration file. Description from CraneFoot v3.2 user's guide: a line of text below a node.

- TracerVariable: Optional. Specified by *NO_DATA* columns in the pedigree file. If used, need to be specified as *<config-file-name>_pedigree.txt* in the configuration file. Description from CraneFoot v3.2 user's guide: square brackets around thename, drawn if integer value greater than 0.

5. The configuration file contains the following formatting and functional parameters:
    - Female
    - FontSize
    - Male
    - PageOrientation
    - PageSize
    - VerboseMode
    - Delimiter
    - BackgroundColor
    - ForegroundColor
    - TimeLimit
    - FigureLimit
    - RandomSeed

    Please refer to CraneFoot v3.2 user's guide for description of these functional parameters.
6. The configuration file is not modified during runs of Genetic ME.
7. Please refer to section 6.1 for an example configuration file.

3.1.2 Pedigree File

1. The pedigree file must be named in the following format: *<config-file-name>-_pedigree.txt*. For example: if the configuration file name is configA.txt, the pedigree file name should be configA_pedigree.txt. It must be in the same directory as the configuration file and the metadata file (if available). In addition, the pedigree file and the directory it is in must be readable and writeable by Genetic ME.
2. CraneFoot was "designed to be compatible with the so called linkage format, where family relations are represented by a set of child-father-mother triplets" (Mäkinen, web access 2011). Its required format for the pedigree file is tabulated text with three columns; each column is associated with a required structural parameter, namely NameVariable, FatherVariable, and MotherVariable. CraneFoot allows those columns associated with the optional parameters listed above to be at a different location (i.e. in a different file other than the pedigree file) and in any order in that file. As such, there is a lot of flexibility creating the pedigree file when CraneFoot is used as a stand-alone application independent from Genetic ME.

3. To ensure a comprehensive merge of pedigree files, Genetic ME imposed the additional requirement that the pedigree file must be a tab-delimited text file containing <u>all</u> columns in the following order:
   - *NAME*: string type
   - *FATHER*: string type; missing values must either be coded as "0" (zero) or end in ".-1"
   - *MOTHER*: string type; missing values must either be coded as "0" (zero) or end in ".-1"

   NOTE: As in LINKAGE-format files, either both parents' names must be specified or both must be missing. It is not allowable to specify only one parent's name – if only one parent is known, you must add a dummy record for the other missing parent. For consistency, if the father's missing ID is coded as "0", the mother's missing ID must also be coded as "0"; similarly, if the father's missing ID is coded to end in ".-1", the mother's missing ID must also be coded to end in ".-1".

   - *GENDER*: “M” or “F”
   - *SIBORD*: integer type
   - *FAMILY*: string type
   - *INDEX*: integer type
   - *NUMBER*: integer type
   - *RISK*: integer type
   - *DISEASE*: integer type
   - *DEAD*: integer type
   - *OCCUPATION*: integer type
   - *GTYPE_A*: string type
   - *GTYPE_B*: string type
   - *NO_DATA*: string type

   The first line of each pedigree file must contain the headings for the columns in the order listed above. At a minimum, each individual’s *NAME, FATHER,* and *MOTHER* fields must contain valid data for the individual to be considered complete. The remaining fields can be left blank. Genetic ME will automatically default any empty string-type field to “ ” and any empty integer-type field to -1.
4. The source pedigree file is preserved during a merge operation. However, if the user chooses to modify the source pedigree such as with an Add New Person or a Remove Person operation, this file will be updated with new information.
5. Please refer to section 6.2 for an example pedigree file.

3.1.3 Metadata File

1. The metadata file is an optional tab-delimited text file with the same number of columns in the same order as for the pedigree file. It is typically generated by Genetic ME to keep track of changes made to the pedigree file during an editing or merging operation. Each row in the metadata file corresponds to a row in the pedigree file and encodes tracking information for the individual of interest. If a metadata file is available from a previous run of Genetic ME, it should be loaded during subsequent run of Genetic ME for continuous tracking of data.
2. The metadata file must be named in the following format: *<config-file-name>-_metadata.txt*. For example: if the configuration file name is configA.txt, the metadata file should be named configA_metadata.txt. In addition, the metadata file must be in the same directory as the configuration and pedigree files. The metadata file and the directory it is in must be readable and writeable by Genetic ME.
3. Depending on user's operation, the metadata file is updated with new tracking information. During a merge operation of two pedigrees, a new metadata file will be created for the resultant merge pedigree to keep track of where the data for an individual originally comes from. This newly created metadata file can be used in subsequent run of Genetic ME to continually update and modify the merge dataset.
4. Please refer to section 6.3 for an example metadata file.

3.2 Output Files

3.2.1 Postscript (*.ps) File

This is generated by CraneFoot and used by Genetic ME to create *.png files. It is accessible outside of Genetic ME application.

3.2.2 Portable Network Graphics (*.png) File

For each *.ps file, three *.png files in sizes small, medium, and large are generated. Once generated, these files are also accessible outside of Genetic ME application.

3.2.3 Metadata (*.txt) File For Source Pedigree

For a source pedigree, if no metadata file is loaded, the application generates one with all the fields defaulted to the name of the source pedigree file.

3.2.4 Merged Dataset

3.2.4.1 Configuration (*.txt) File

A new configuration file "Merged.txt" is generated for each merge. It is accessible outside of Genetic ME at the directory containing the "to" pedigree specified during the merge if you choose to save the merge dataset using File → Save to File → Merged Tree.

3.2.4.2 Pedigree (*.txt) File

A new pedigree file "Merged_pedigree.txt" is generated for each merge. It is accessible outside of Genetic ME at the directory containing the "to" pedigree specified during the merge if you choose to save the merge dataset using File → Save to File → Merged Tree.

3.2.4.3 Metadata (*.txt) File

A new metadata file "Merged_metadata.txt" is generated for each merge. It is accessible outside of Genetic ME at the directory containing the "to" pedigree specified during the merge if you choose to save the merge dataset using File → Save to File → Merged Tree.

3.2.4.4 Graphic (*.ps and *.png) Files

A *.ps file and three *.png (sizes small, medium, and large) files are created and accessible outside of Genetic ME at the directory containing the "to" pedigree specified during the merge if you choose to save the merge dataset using File → Save to File → Merged Tree.

4 Basic uses

4.1 Starting Genetic ME

- Double click on GeneticME.jar to launch the main window.

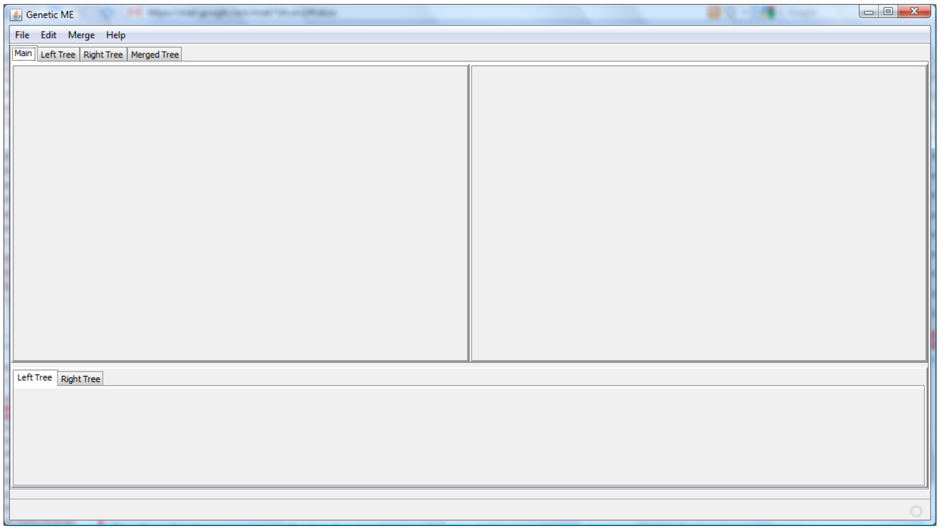

4.2 Opening Pedigree Files

- File → Open or Ctrl + O

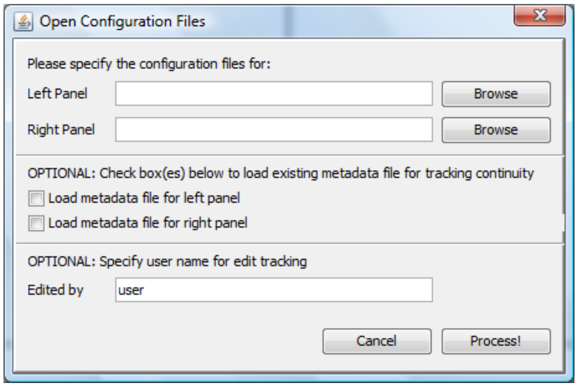

- Click on “Browse” buttons to specify the configuration files for left panel and right panel. Check the appropriate box(es) to load the metadata file(s) for continuous tracking. If left panel box is unchecked, new metadata file(s) will be created. Specify the name of the user so all changes made during this session can be tracked to the current user. Note: if you choose to create a new metadata file, it will replace any existing file with the name <*config-file-name*>_metadata.txt in the current directory.

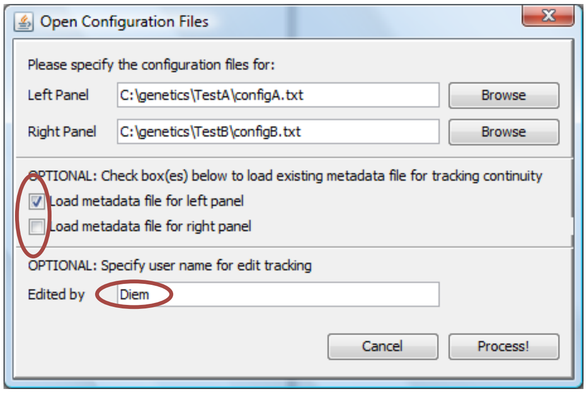

- Click “Process!”

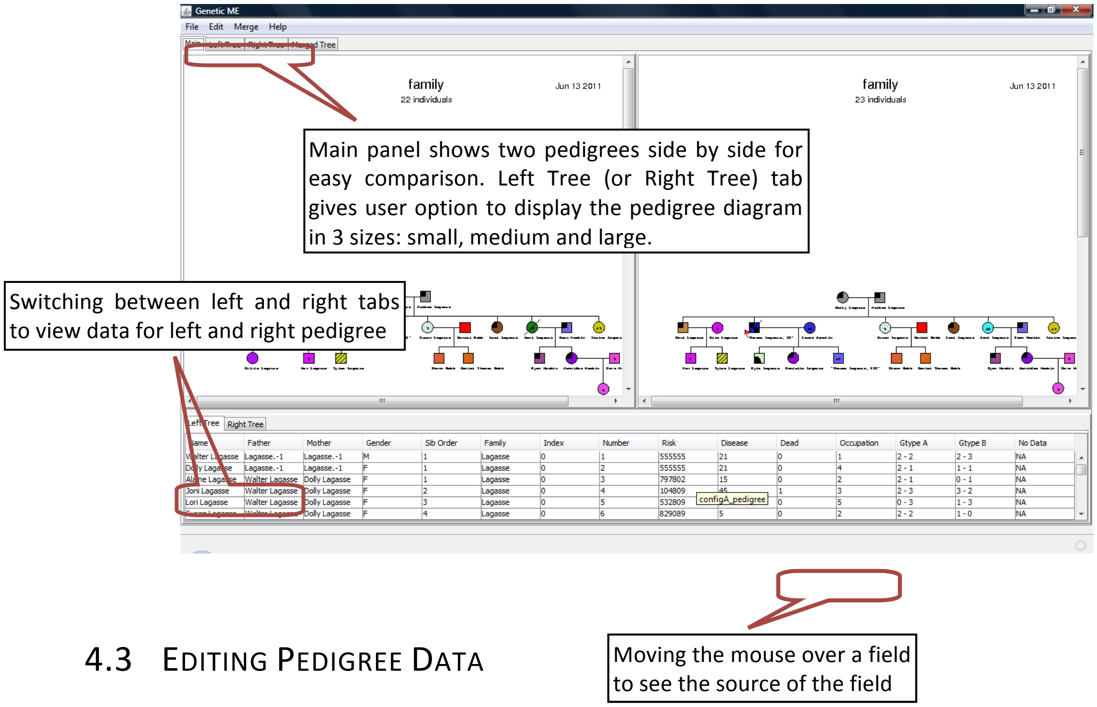

4.3 Editing Pedigree Data

4.3.1 By manually Changing the Data in the Table

- Place the cursor over the field you would like to change and double-click. Erase the old value and type in a new value.
- Preview the changes you have made by choosing Edit → Preview Change(s) → Left (or Right) Tree. Note: changes are not saved to a file and would be lost if you quit the application at this point.
- If you are satisfied with the new changes, and would like to save them to a file, do File → Save to File → Left (or Right) Tree. This will overwrite the original source pedigree and metadata files. The updated data field(s) will be denoted as “Edited by <user>”. Note that once you save the new file, you cannot recover the old file (unless you have made a copy of the original file).
- If you are not satisfied with the new changes, and would like to revert to the old file, do File → Reload → Left (or Right) Tree. This will redisplay the most recently saved file saved before the new changes are made.

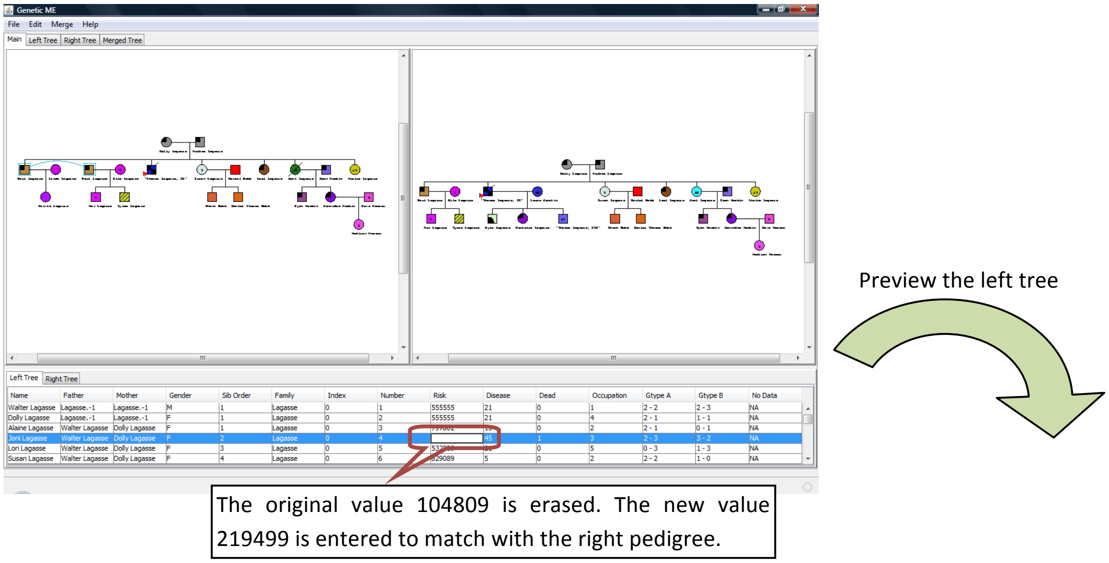

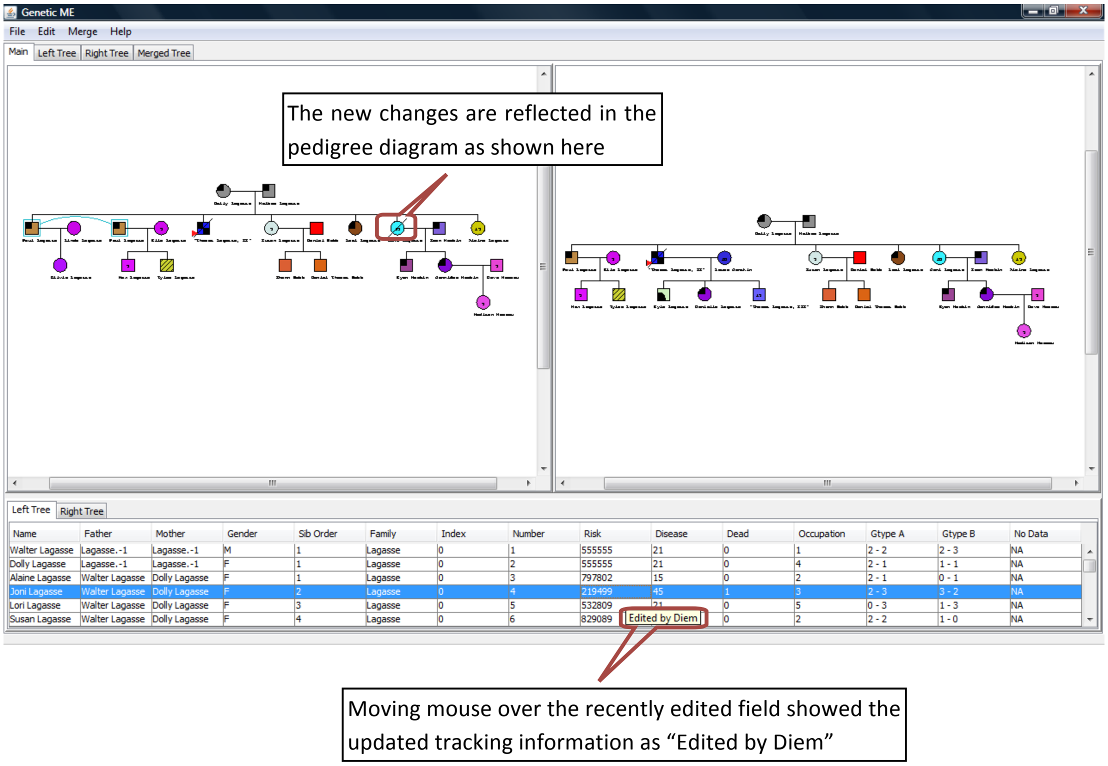

4.3.2 By Adding a New Person

- Display the “add person(s)” window by choosing Edit → Add Person → Left (or Right) Tree

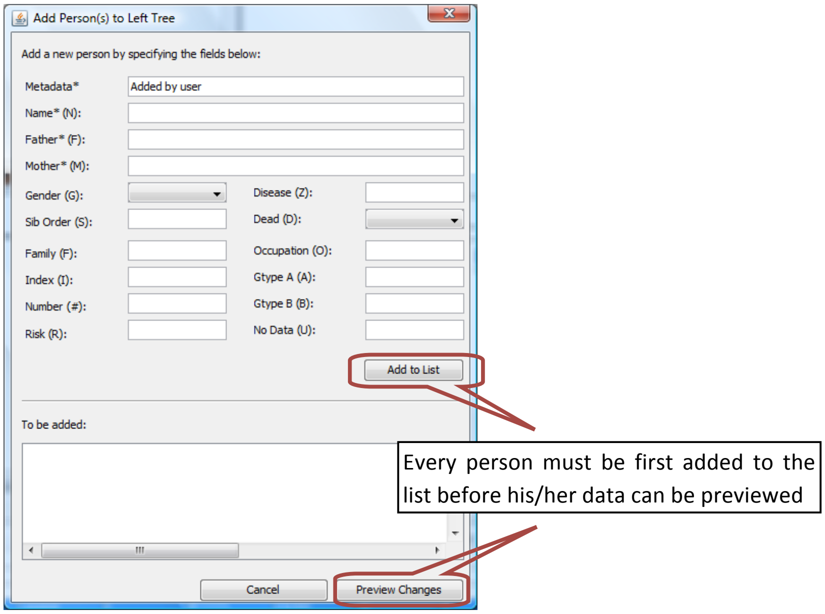

- Fill out the appropriate fields, then click "Add to List" button. You can add multiple individuals at once by adding them to the list. Once you're done adding new persons to list, click "Preview Changes" to view the updated pedigree diagram. Please note that you need to add a person to the list before that person can be added to the diagram.
- If you are satisfied with the new changes and would like to save them to file, do File → Save to File → Left (or Right) Tree. This will overwrite the original source pedigree and metadata file. The new data will be denoted as "Added by <user>". Once you save a new file, you cannot recover the old file (unless you have made a copy of the original file).
- If you are not satisfied with the new changes and would like to revert to the old file, do File → Reload → Left (or Right) Tree. This will redisplay the most recently saved file before the new changes are made.

4.3.2.1 By Removing an Existing Person

- Display the "remove person(s)" window by Edit → Remove Person(s) → Left (or Right) Tree. Choose a person to be removed from the drop down list. The application will automatically fill in all the fields with the appropriate data for the given person from the source pedigree file.

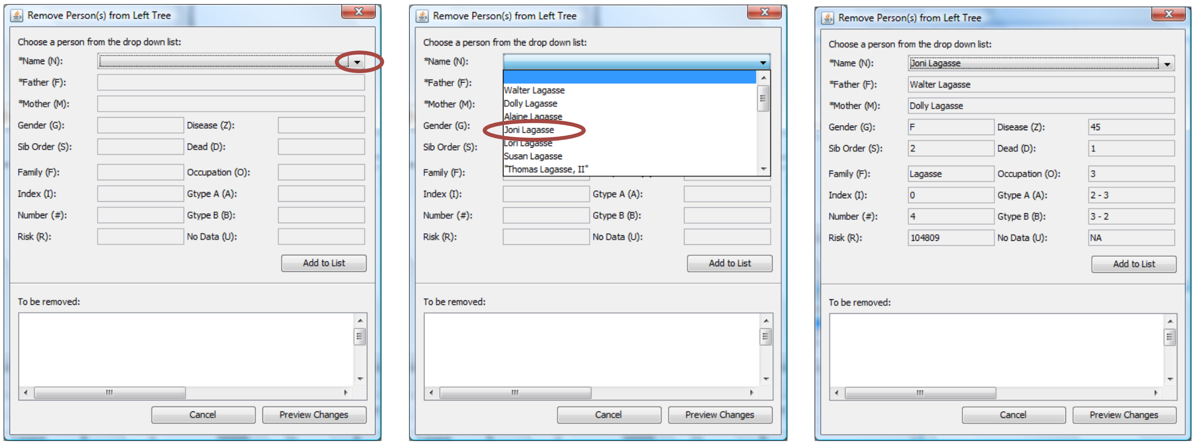

- Click "Add to List" button. You can remove multiple individuals at once by adding all them to the list. Once you are done adding persons to be removed to the list, click "Preview Changes" to view the updated pedigree diagram. Please note that you need to add a person to the list before that person can be removed from the diagram. Caution should be exercised when you are removing persons within a pedigree in terms of the following aspects:
    - Not all persons can be freely removed from a pedigree. It would be an error if the person to be removed contributed to the core structure of the pedigree. For example, consider the pedigree below – if one tries to only remove "Mary Boleyn", this will result in an error because Mary Boleyn would still be listed as the mother of Catherine Carey and Henry Carey.

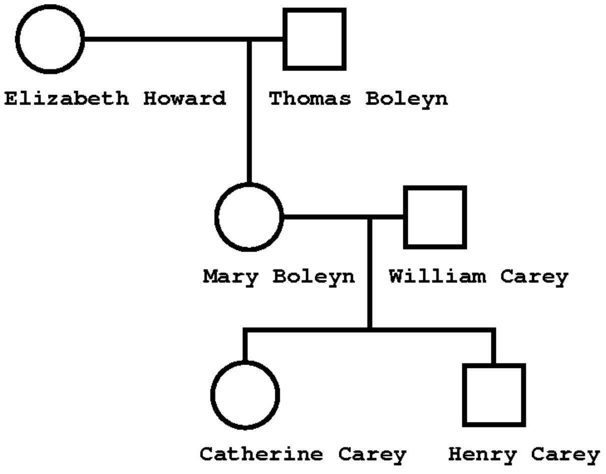

    - In the current version, users cannot modify the add-person list after adding a person. If you wish to remove any person from the list, you would have to delete

him or her by clicking on the “cancel” button, and then restart the removal manipulation process.
    - If you repeatedly selected the same person to be removed, the list would include duplicates.
- If you are satisfied with the new changes, and would like to save them to file, do File → Save to File → Left (or Right) Tree. The person(s) listed will be removed from the original source pedigree and metadata file. Once you save a new file, you cannot recover the old file (unless you have made a copy of the original file).
- If you are not satisfied with the new changes, and would like to revert to the old file: File → Reload → Left (or Right) Tree. This will redisplay the most recently saved file before the new changes are made.

4.3.3 By Reconciling Attributes

- Display the “reconciling records” window by choosing Merge → Record Reconciliation. This window contains a drop-down list of all individuals that exist in both source pedigrees. Individuals are determined to be the same if they have the same spelling for the *NAME* field. Those that exist in one pedigree but not the other are not displayed in this drop-down list.
- When you choose an individual from the drop-down list, attributes for the individual from each of the (saved) source pedigree are displayed. Those attributes that are the same in both pedigrees are displayed in black with disabled radio-buttons. Those attributes with discrepancy are highlighted in red with enabled radio-buttons to allow you to choose which version is correct.
- Specify which source pedigree file you would like to save to by checking/un-checking the “Save to left pedigree” or “Save to right pedigree” box.
- Click “Preview Changes” to see the updated pedigree diagram.
- If you are satisfied with the new changes, and would like to save them to file, do File → Save to File → Left (or Right) Tree. The updated attribute fields will be tagged as coming from the other source pedigree file. Once you save a new file, you cannot recover the old file (unless you have made a copy of the original file).
- If you are not satisfied with the new changes and would like to revert to the old file, do File → Reload → Left (or Right) Tree. This will redisplay the most recently saved file before the new changes are made.

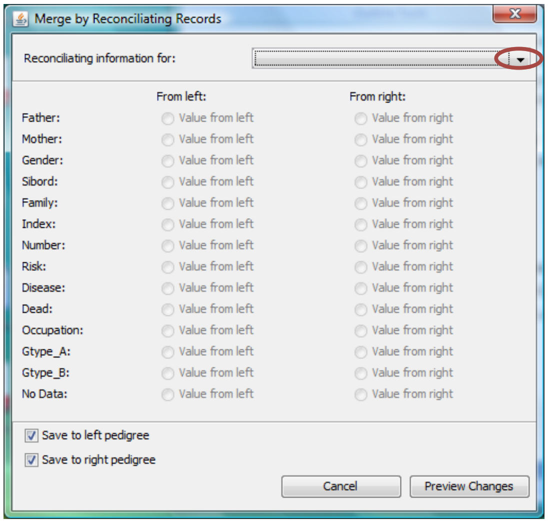

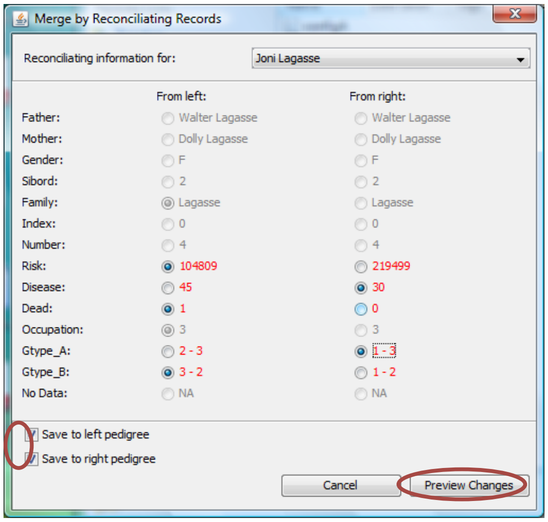

4.4 Merging Pedigrees

For the purpose of merging, all the algorithms below consider two persons to be the same if they have the same *NAME* field regardless of their attributes. During the merging process, the algorithms retain the version of the person in the destination pedigree file.

For example, Joni Lagasse is considered to be the same person in both pedigree A and B, despite the fact that several of her attributes are listed differently in pedigree A as compared to pedigree B. When merging from pedigree A to pedigree B, the version of Joni Lagasse in pedigree B will be saved in the final merged pedigree. If you are concerned about the discrepancies, it is best to reconcile all differences by using the "Reconciliation Records" window before you perform a merge.

	Name	...	Risk	Disease	Dead	Occupation	Gtype_A	GType_B	No_Data
Pedigree A	Joni Lagasse	...	104809	45	1	3	2 – 3	3 – 2	NA
Pedigree B	Joni Lagasse	...	219499	30	0	3	1 – 3	1 – 2	NA

4.4.1 By Combining Data from One Pedigree into Another

- Display "the merge by combination" window by choosing Merge → Combination.
- Specify the direction of the merge ("Left Tree to Right Tree" *or* "Right Tree to Left Tree"). For example, if "Left Tree to Right Tree" is chosen, the left tree will be the source pedigree, and right tree the destination pedigree. The application updates the two drop-down lists with the names of individuals that appear in both the left pedigree and right pedigree.

- Choose the same individual whose descendants are to be combined from both drop-down lists and click "Go!" The application will display the new merged pedigree diagram in the "Merged Pedigree" tab in the main window.
- If you choose to save the merged dataset (File → Save to File → Merged Tree), all files associated with this merging manipulation (configuration, pedigree, metadata, and graphic files) are stored in the same directory as the destination pedigree.

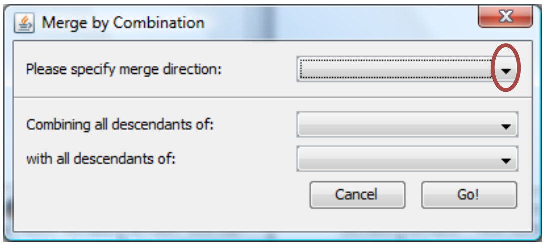

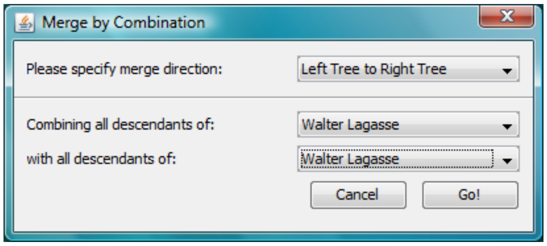

Example of merging by combination: Here we start with a "left" pedigree of three people, and a "right" pedigree of seven people:

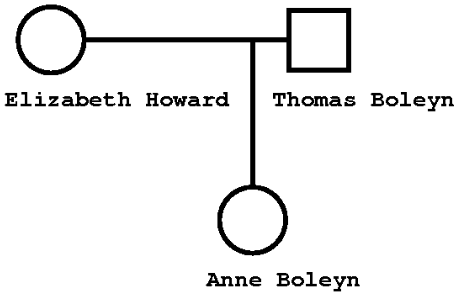

Left pedigree

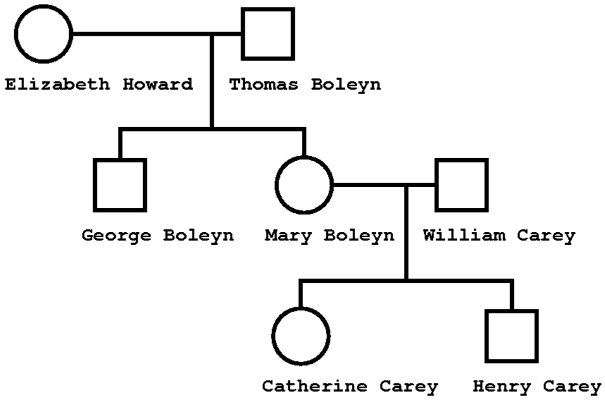

Right pedigree

This Merge by Combination:

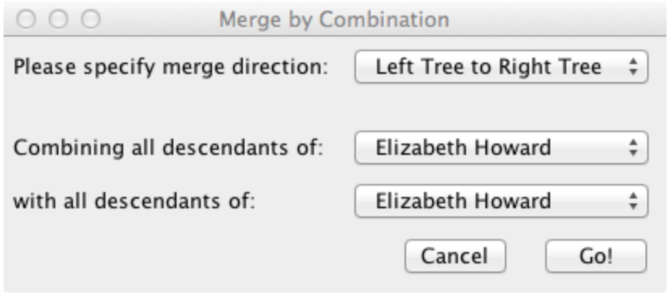

results in this merged pedigree containing eight people, where Anne Boleyn has been added in:

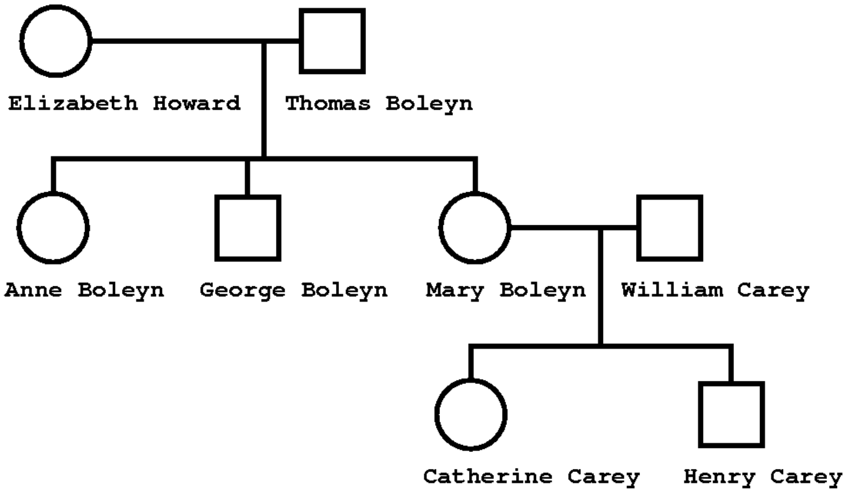

4.4.2 By Replacing an Individual or Branch from One Pedigree by the Other

- Display the "merge by replacement" window by choosing Merge → Replacement.
- Specify the direction of the merge ("Left Tree to Right Tree" *or* "Right Tree to Left Tree"). The application updates the two drop-down lists with the names of individuals in the appropriate pedigree.
- Make the appropriate choice and click "Go!" The application displays the new merged pedigree diagram in the "Merged Pedigree" tab in the main window.
- If you choose to save the merged dataset (File → Save to File → Merged Tree), all files associated with this merging manipulation (configuration, pedigree, metadata, and graphic files) are stored in the same directory as the destination pedigree.

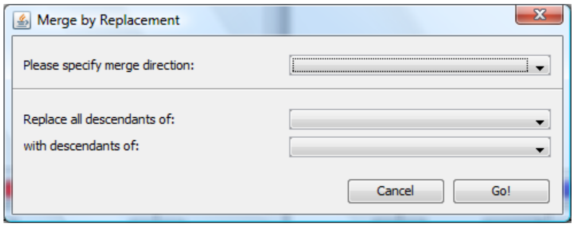

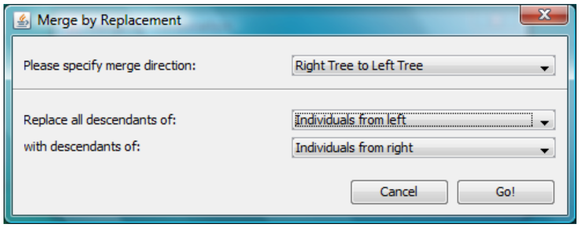

Examples of merging by replacement: Here we start with a "left" pedigree of three people, and a "right" pedigree of seven people:

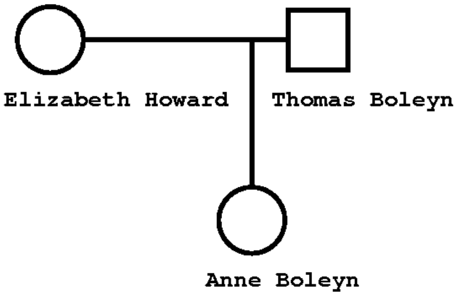

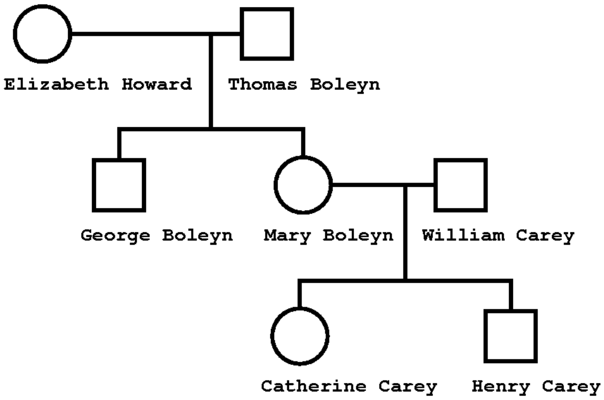

Left pedigree **Right pedigree**

This Merge by Replacement:

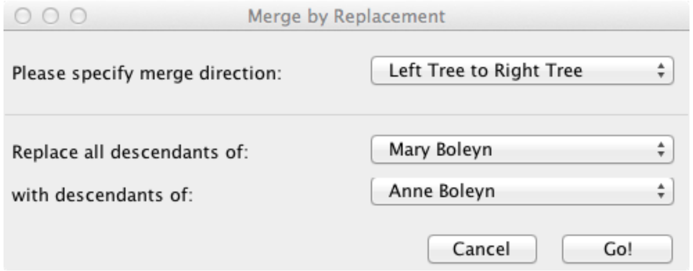

results in this merged pedigree, where Mary Boleyn and her descendants have been replaced with Anne Boleyn (and her non-existent descendants):

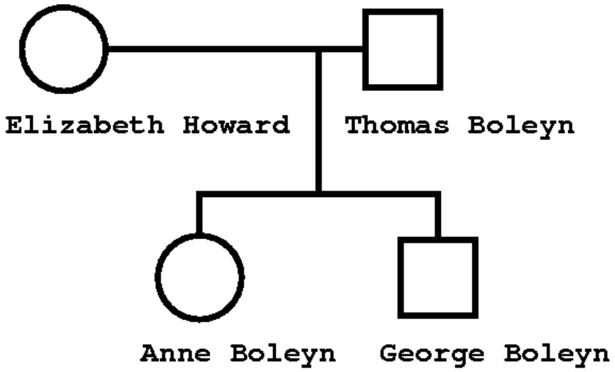

In contrast, this Merge by Replacement:

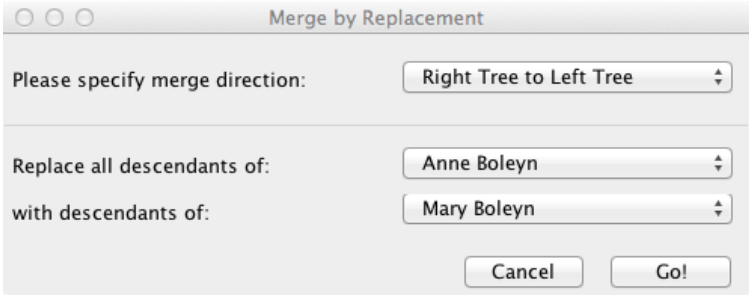

results in this merged pedigree, where Anne Boleyn has been replaced by Mary Boleyn and her descendants:

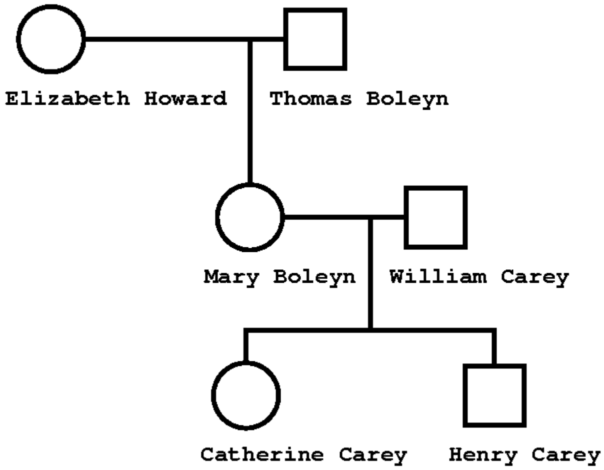

4.4.3 By Adding Individuals and Sublineages from One Pedigree to the Other

- Display the "merge by addition" window by choosing Merge → Addition.
- Specify the direction of the merge ("Left Tree to Right Tree" *or* "Right Tree to Left Tree"). The application updates the two drop-down lists with the names of individuals in the appropriate pedigree.
- Make the appropriate choice and click "Go!" The application displays the new merged pedigree diagram in the "Merged Pedigree" tab in the main window.
- If you choose to save the merged dataset (File → Save to File → Merged Tree), all files associated with this merging manipulation (configuration, pedigree, metadata, and graphic files) are stored in the same directory as the destination pedigree.

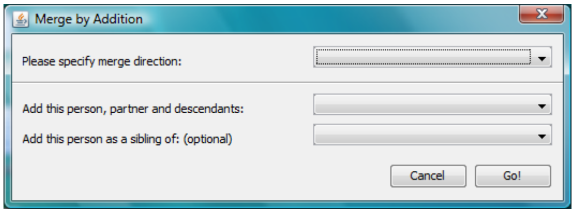

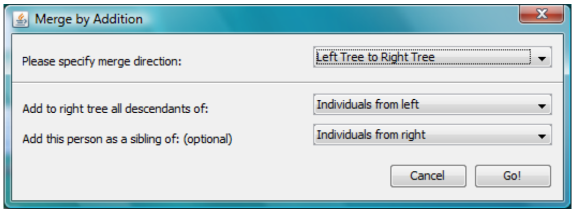

Examples of merging by addition: Here we start with a "left" pedigree of three people, and a "right" pedigree of 7 people:

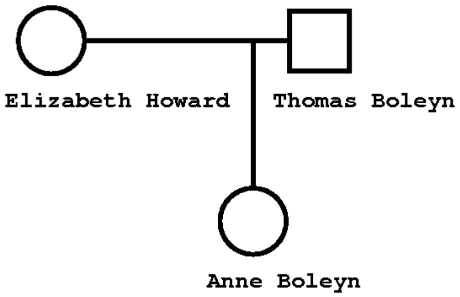

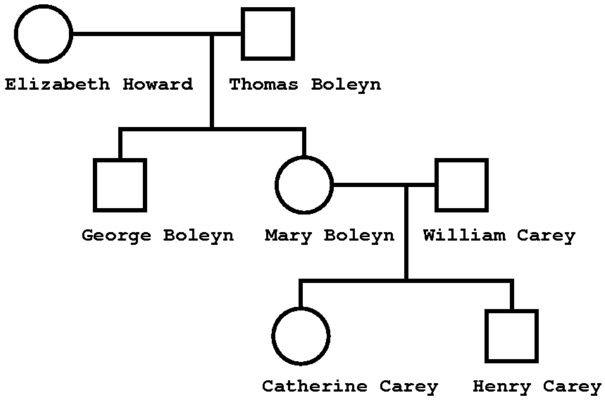

Left pedigree **Right pedigree**

This Merge by Addition:

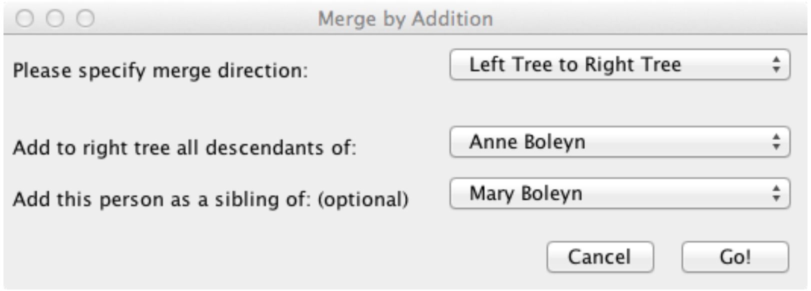

results in this merged pedigree, where Anne Boleyn has been added as a sibling of Mary Boleyn:

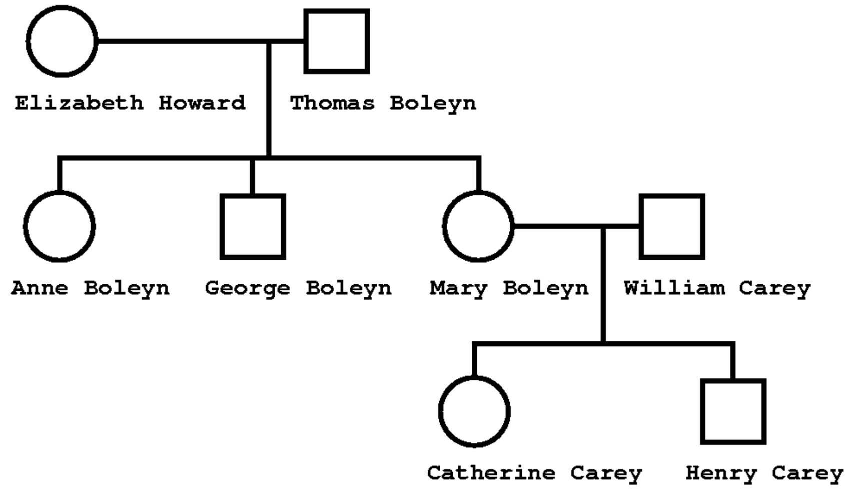

This Merge by Addition:

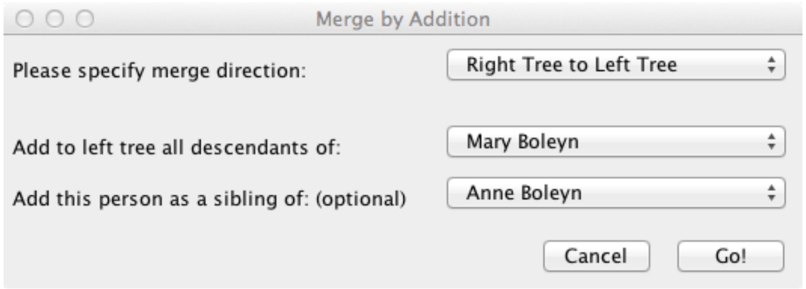

results in this merged pedigree, where Mary Boleyn and her descendants have been added:

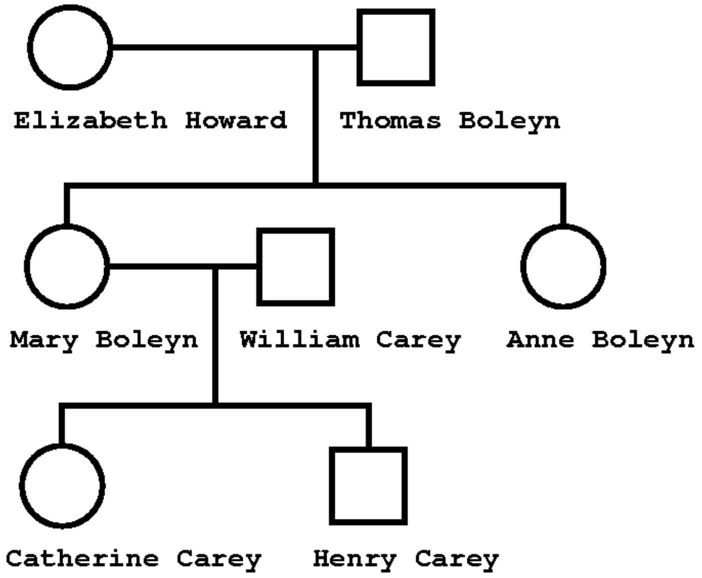

5 REFERENCES

1. Mäkinen VP, Parkkonen M, Wessman M, et al. High-throughput pedigree drawing. *European Journal of Human Genetics*. 2005 Aug; 13(8):987-9.
2. Mäkinen VP. CraneFoot v3.2 user's guide. <http://www.finndiane.fi/software/cranefoot/guide.pdf>. Web. Jun 15, 2011.

6 APPENDIX

The example input files for the larger Lagasse pedigrees used above can be found in the "example_data/example1" folder of the Genetic ME distribution, while example input files for the smaller Boleyn pedigrees used above can be found in the "example_data/example2" folder. Examples from the example1 Lagasse set of files are shown below:

6.1 EXAMPLE CONFIGURATION FILE

```
# Cranefoot3 configuration file.
# Original file created by Ville-Petteri Makinen 2006
# Adapted by Diem Bui 2011 for use with Genetic ME

# Use these two instructions to specify the input file and naming of the output file. If this
# configuration file is named "configA.txt", then the PedigreeFile should be set to
# "configA_pedigree.txt" and the PedigreeName to "configA_results".
PedigreeFile        configA_pedigree.txt
PedigreeName     configA_results

# Structural parameters
```

```
# Name, father and mother are defaulted to the PedigreeFile by CraneFoot. Since CraneFoot
# allows the rest of the variable to come from a different file but Genetic ME enforces them to
# be in the PedigreeFile, you need to explicitly specify the source for the latter variables if you
# plan to use them.
NameVariable          NAME
FatherVariable        FATHER
MotherVariable        MOTHER
#SubgraphVariable     FAMILY      configA_pedigree.txt
GenderVariable        GENDER      configA_pedigree.txt
AgeVariable           SIBORD      configA_pedigree.txt

# Visualization parameters
ArrowVariable         INDEX       configA_pedigree.txt
ColorVariable         RISK        configA_pedigree.txt
PatternVariable       DISEASE     configA_pedigree.txt
SlashVariable         DEAD        configA_pedigree.txt
#ShapeVariable        OCCUP       configA_pedigree.txt
TextVariable          NAME        configA_pedigree.txt
#TextVariable         GTYPE_A     configA_pedigree.txt
#TextVariable         GTYPE_B     configA_pedigree.txt
#TracerVariable       NO_DATA     configA_pedigree.txt

# It is often necessary to list the meanings of symbols so that every reader can understand the
# pedigree picture. CraneFoot create a legend for every family automatically, based on the
# common instructions. These info commands do not affect the pedigree itself in any way,
# except taking a small portion of the page. The first value (e.g. 'low') is a short description of
# the symbol, and the second (e.g. 999900) indicates the symbol itself.
#ColorInfo     low         999900
#ColorInfo     high        000099
#PatternInfo   mild        21
#PatternInfo   moderate    41
#PatternInfo   severe      52
#ShapeInfo     student     1
#ShapeInfo     sailor      2
#ShapeInfo     scientist   3
#ShapeInfo     unknown     4
#ShapeInfo     prisoner    5
#ShapeInfo     pensioner   6
#ShapeInfo     athlete     7
#ShapeInfo     priest      8

# Unlike the previous versions of CraneFoot, the third generation employs an undeterministic
# optimization algorithm the spreads the family graph around the canvas. For this reason, the
# user can set a time limit to ensure that the program completes in reasonable time. It is also
# possible to set a fixed seed for the random number generator to ensure repeatable layouts.
#RandomSeed    12345
#TimeLimit     30

# Miscellaneous commands. The first value for PaperSize sets the main document dimensions,
# the second sets a fixed paper size for the .eps files. The optimal bounding box for an .eps file
# is rarely a standard paper size and thus might cause problems when converting to other
# formats.
#Delimiter            tab              # tab/ws/(character)
#FigureLimit          10               # max number of .eps files
#FontSize             10               # pt
#BackgroundColor      999999           # RRGGBB
#ForegroundColor      000000
PageSize              letter   auto    # a0...a5/letter/auto
PageOrientation       portrait         # portrait/landscape
#VerboseMode          on               # on/off
```

6.2 Example Pedigree File

In this example, note that when the father and mother names are unknown, they both are coded to end in ".-1".

NAME	FATHER	MOTHER	GENDER	SIBORD	FAMILY	INDEX	NUMBER	RISK	DISEASE	DEAD	OCCUP	GTYPE_A	GTYPE_B	NO_DATA
**Walter Lagasse**	Lagasse.-1	Lagasse.-1	M	1	Lagasse	0	1	555555	21	0	1	2 - 2	2 - 3	NA
**Dolly Lagasse**	Lagasse.-1	Lagasse.-1	F	1	Lagasse	0	2	555555	21	0	4	2 - 1	1 - 1	NA
**Alaine Lagasse**	Walter Lagasse	Dolly Lagasse	F	1	Lagasse	0	3	797802	15	0	2	2 - 1	0 - 1	NA
**Joni Lagasse**	Walter Lagasse	Dolly Lagasse	F	2	Lagasse	0	4	104809	45	1	3	2 - 3	3 - 2	NA
**Lori Lagasse**	Walter Lagasse	Dolly Lagasse	F	3	Lagasse	0	5	532809	21	0	5	0 - 3	1 - 3	NA
**Susan Lagasse**	Walter Lagasse	Dolly Lagasse	F	4	Lagasse	0	6	829089	5	0	2	2 - 2	1 - 0	NA
**Thomas Lagasse, II**	Walter Lagasse	Dolly Lagasse	M	5	Lagasse	1	7	111199	52	1	4	2 - 1	1 - 1	NA
**Paul Lagasse**	Walter Lagasse	Dolly Lagasse	M	6	Lagasse	0	8	735326	21	0	2	2 - 1	0 - 1	NA
**Daniel Hebb**	Hebb.-1	Hebb.-1	M	1	Hebb	0	9	990000	1	0	2	1 - 3	1 - 2	NA
**Daniel T Hebb**	Daniel Hebb	Susan Lagasse	M	1	Hebb	0	10	853908	1	0	2	0 - 3	1 - 3	NA
**Shawn Hebb**	Daniel Hebb	Susan Lagasse	M	2	Hebb	0	11	853720	1	0	5	2 - 2	1 - 0	NA
**Ella Lagasse**	Lagasse.-1	Lagasse.-1	F	1	Lagasse	0	12	810492	5	0	1	2 - 2	2 - 3	NA
**Tyler Lagasse**	Paul Lagasse	Ella Lagasse	M	1	Lagasse	0	13	798219	11	0	4	2 - 1	1 - 1	NA
**Max Lagasse**	Paul Lagasse	Ella Lagasse	M	2	Lagasse	0	14	860798	5	0	2	2 - 1	0 - 1	NA
**Sean Martin**	Martin.-1	Martin.-1	M	1	Martin	0	15	493784	21	0	3	1 - 3	1 - 2	NA
**Jennifer Martin**	Sean Martin	Joni Lagasse	F	1	Martin	0	16	510483	21	0	5	0 - 3	1 - 3	NA
**Ryan Martin**	Sean Martin	Joni Lagasse	M	2	Martin	0	17	602758	21	0	2	1 - 3	2 - 1	NA
**Dave Moreau**	Moreau.-1	Moreau.-1	M	1	Moreau	0	18	902783	5	0	4	0 - 2	1 - 2	NA
**Madison Moreau**	Dave Moreau	Jennifer Martin	F	1	Moreau	0	19	893089	5	0	2	1 - 1	2 - 1	NA
**Linda Lagasse**	Lagasse.-1	Lagasse.-1	F	1	Lagasse	0	20	780389	1	0	2	2 -1	1 - 0	NA
**Olivia Lagasse**	Paul Lagasse	Linda Lagasse	F	1	Lagasse	0	21	690798	1	0	2	2 - 0	0 - 1	NA

Note: This file should be in tab-delimited text file. It is shown here in table format for the ease of reading.

6.3 Example Metadata File (as generated by Genetic ME)

NAME	FATHER	MOTHER	GENDER	SIBORD	FAMILY	INDEX	NUMBER	RISK	DISEASE	DEAD	OCCUP	GTYPE_A	GTYPE_B	NO_DATA
configA_pedigree	configA_pedigree	configA_pedigree	configA_pedigree	configA_pedigree	configA_pedigree	configA_pedigree	configA_pedigree	configA_pedigree	configA_pedigree	configA_pedigree	configA_pedigree	configA_pedigree	configA_pedigree	configA_pedigree
configA_pedigree	configA_pedigree	configA_pedigree	configA_pedigree	configA_pedigree	configA_pedigree	configA_pedigree	configA_pedigree	configA_pedigree	configA_pedigree	configA_pedigree	configA_pedigree	configA_pedigree	configA_pedigree	configA_pedigree
configA_pedigree	configA_pedigree	configA_pedigree	configA_pedigree	configA_pedigree	configA_pedigree	configA_pedigree	configA_pedigree	configA_pedigree	configA_pedigree	configA_pedigree	configA_pedigree	configA_pedigree	configA_pedigree	configA_pedigree
configA_pedigree	configA_pedigree	configA_pedigree	configA_pedigree	configA_pedigree	configA_pedigree	configA_pedigree	configA_pedigree	configA_pedigree	configA_pedigree	configA_pedigree	configA_pedigree	configA_pedigree	configA_pedigree	configA_pedigree
configA_pedigree	configA_pedigree	configA_pedigree	configA_pedigree	configA_pedigree	configA_pedigree	configA_pedigree	configA_pedigree	configA_pedigree	configA_pedigree	configA_pedigree	configA_pedigree	configA_pedigree	configA_pedigree	configA_pedigree
configA_pedigree	configA_pedigree	configA_pedigree	configA_pedigree	configA_pedigree	configA_pedigree	configA_pedigree	configA_pedigree	configA_pedigree	configA_pedigree	configA_pedigree	configA_pedigree	configA_pedigree	configA_pedigree	configA_pedigree
configA_pedigree	configA_pedigree	configA_pedigree	configA_pedigree	configA_pedigree	configA_pedigree	configA_pedigree	configA_pedigree	configA_pedigree	configA_pedigree	configA_pedigree	configA_pedigree	configA_pedigree	configA_pedigree	configA_pedigree
configA_pedigree	configA_pedigree	configA_pedigree	configA_pedigree	configA_pedigree	configA_pedigree	configA_pedigree	configA_pedigree	configA_pedigree	configA_pedigree	configA_pedigree	configA_pedigree	configA_pedigree	configA_pedigree	configA_pedigree
configA_pedigree	configA_pedigree	configA_pedigree	configA_pedigree	configA_pedigree	configA_pedigree	configA_pedigree	configA_pedigree	configA_pedigree	configA_pedigree	configA_pedigree	configA_pedigree	configA_pedigree	configA_pedigree	configA_pedigree
configA_pedigree	configA_pedigree	configA_pedigree	configA_pedigree	configA_pedigree	configA_pedigree	configA_pedigree	configA_pedigree	configA_pedigree	configA_pedigree	configA_pedigree	configA_pedigree	configA_pedigree	configA_pedigree	configA_pedigree
configA_pedigree	configA_pedigree	configA_pedigree	configA_pedigree	configA_pedigree	configA_pedigree	configA_pedigree	configA_pedigree	configA_pedigree	configA_pedigree	configA_pedigree	configA_pedigree	configA_pedigree	configA_pedigree	configA_pedigree
configA_pedigree	configA_pedigree	configA_pedigree	configA_pedigree	configA_pedigree	configA_pedigree	configA_pedigree	configA_pedigree	configA_pedigree	configA_pedigree	configA_pedigree	configA_pedigree	configA_pedigree	configA_pedigree	configA_pedigree
configA_pedigree	configA_pedigree	configA_pedigree	configA_pedigree	configA_pedigree	configA_pedigree	configA_pedigree	configA_pedigree	configA_pedigree	configA_pedigree	configA_pedigree	configA_pedigree	configA_pedigree	configA_pedigree	configA_pedigree
configA_pedigree	configA_pedigree	configA_pedigree	configA_pedigree	configA_pedigree	configA_pedigree	configA_pedigree	configA_pedigree	configA_pedigree	configA_pedigree	configA_pedigree	configA_pedigree	configA_pedigree	configA_pedigree	configA_pedigree
configA_pedigree	configA_pedigree	configA_pedigree	configA_pedigree	configA_pedigree	configA_pedigree	configA_pedigree	configA_pedigree	configA_pedigree	configA_pedigree	configA_pedigree	configA_pedigree	configA_pedigree	configA_pedigree	configA_pedigree
configA_pedigree	configA_pedigree	configA_pedigree	configA_pedigree	configA_pedigree	configA_pedigree	configA_pedigree	configA_pedigree	configA_pedigree	configA_pedigree	configA_pedigree	configA_pedigree	configA_pedigree	configA_pedigree	configA_pedigree
configA_pedigree	configA_pedigree	configA_pedigree	configA_pedigree	configA_pedigree	configA_pedigree	configA_pedigree	configA_pedigree	configA_pedigree	configA_pedigree	configA_pedigree	configA_pedigree	configA_pedigree	configA_pedigree	configA_pedigree
configA_pedigree	configA_pedigree	configA_pedigree	configA_pedigree	configA_pedigree	configA_pedigree	configA_pedigree	configA_pedigree	configA_pedigree	configA_pedigree	configA_pedigree	configA_pedigree	configA_pedigree	configA_pedigree	configA_pedigree
configA_pedigree	configA_pedigree	configA_pedigree	configA_pedigree	configA_pedigree	configA_pedigree	configA_pedigree	configA_pedigree	configA_pedigree	configA_pedigree	configA_pedigree	configA_pedigree	configA_pedigree	configA_pedigree	configA_pedigree
configA_pedigree	configA_pedigree	configA_pedigree	configA_pedigree	configA_pedigree	configA_pedigree	configA_pedigree	configA_pedigree	configA_pedigree	configA_pedigree	configA_pedigree	configA_pedigree	configA_pedigree	configA_pedigree	configA_pedigree
configA_pedigree	configA_pedigree	configA_pedigree	configA_pedigree	configA_pedigree	configA_pedigree	configA_pedigree	configA_pedigree	configA_pedigree	configA_pedigree	configA_pedigree	configA_pedigree	configA_pedigree	configA_pedigree	configA_pedigree

7 License Agreement

Genetic ME is licensed under the GNU General Public License Version 3, which reads as follows:

GNU GENERAL PUBLIC LICENSE

Version 3, 29 June 2007

Copyright © 2007 Free Software Foundation, Inc. <http://fsf.org/>

Everyone is permitted to copy and distribute verbatim copies of this license document, but changing it is not allowed.

Preamble

The GNU General Public License is a free, copyleft license for software and other kinds of works.

The licenses for most software and other practical works are designed to take away your freedom to share and change the works. By contrast, the GNU General Public License is intended to guarantee your freedom to share and change all versions of a program--to make sure it remains free software for all its users. We, the Free Software Foundation, use the GNU General Public License for most of our software; it applies also to any other work released this way by its authors. You can apply it to your programs, too.

When we speak of free software, we are referring to freedom, not price. Our General Public Licenses are designed to make sure that you have the freedom to distribute copies of free software (and charge for them if you wish), that you receive source code or can get it if you want it, that you can change the software or use pieces of it in new free programs, and that you know you can do these things.

To protect your rights, we need to prevent others from denying you these rights or asking you to surrender the rights. Therefore, you have certain responsibilities if you distribute copies of the software, or if you modify it: responsibilities to respect the freedom of others.

For example, if you distribute copies of such a program, whether gratis or for a fee, you must pass on to the recipients the same freedoms that you received. You must make sure that they, too, receive or can get the source code. And you must show them these terms so they know their rights.

Developers that use the GNU GPL protect your rights with two steps: (1) assert copyright on the software, and (2) offer you this License giving you legal permission to copy, distribute and/or modify it.

For the developers' and authors' protection, the GPL clearly explains that there is no warranty for this free software. For both users' and authors' sake, the GPL requires that modified versions be marked as changed, so that their problems will not be attributed erroneously to authors of previous versions.

Some devices are designed to deny users access to install or run modified versions of the software inside them, although the manufacturer can do so. This is fundamentally incompatible with the aim of protecting users' freedom to change the software. The systematic pattern of such abuse occurs in the area of products for individuals to use, which is precisely where it is most unacceptable. Therefore, we have designed this version of the GPL to prohibit the practice for those products. If such problems arise substantially in other domains, we stand ready to extend this provision to those domains in future versions of the GPL, as needed to protect the freedom of users.

Finally, every program is threatened constantly by software patents. States should not allow patents to restrict development and use of software on general-purpose computers, but in those that do, we wish to avoid the special danger that patents applied to a free program could make it effectively proprietary. To prevent this, the GPL assures that patents cannot be used to render the program non-free.

The precise terms and conditions for copying, distribution and modification follow.

TERMS AND CONDITIONS

0. Definitions.

“This License” refers to version 3 of the GNU General Public License.

“Copyright” also means copyright-like laws that apply to other kinds of works, such as semiconductor masks.

“The Program” refers to any copyrightable work licensed under this License. Each licensee is addressed as “you”. “Licensees” and “recipients” may be individuals or organizations.

To “modify” a work means to copy from or adapt all or part of the work in a fashion requiring copyright permission, other than the making of an exact copy. The resulting work is called a “modified version” of the earlier work or a work “based on” the earlier work.

A “covered work” means either the unmodified Program or a work based on the Program.

To “propagate” a work means to do anything with it that, without permission, would make you directly or secondarily liable for infringement under applicable copyright law, except executing it on a computer or modifying a private copy. Propagation includes copying, distribution (with or without modification), making available to the public, and in some countries other activities as well.

To “convey” a work means any kind of propagation that enables other parties to make or receive copies. Mere interaction with a user through a computer network, with no transfer of a copy, is not conveying.

An interactive user interface displays “Appropriate Legal Notices” to the extent that it includes a convenient and prominently visible feature that (1) displays an appropriate copyright notice, and (2) tells the user that there is no warranty for the work (except to the extent that warranties are provided), that

licensees may convey the work under this License, and how to view a copy of this License. If the interface presents a list of user commands or options, such as a menu, a prominent item in the list meets this criterion.

1. Source Code.

The “source code” for a work means the preferred form of the work for making modifications to it. “Object code” means any non-source form of a work.

A “Standard Interface” means an interface that either is an official standard defined by a recognized standards body, or, in the case of interfaces specified for a particular programming language, one that is widely used among developers working in that language.

The “System Libraries” of an executable work include anything, other than the work as a whole, that (a) is included in the normal form of packaging a Major Component, but which is not part of that Major Component, and (b) serves only to enable use of the work with that Major Component, or to implement a Standard Interface for which an implementation is available to the public in source code form. A “Major Component”, in this context, means a major essential component (kernel, window system, and so on) of the specific operating system (if any) on which the executable work runs, or a compiler used to produce the work, or an object code interpreter used to run it.

The “Corresponding Source” for a work in object code form means all the source code needed to generate, install, and (for an executable work) run the object code and to modify the work, including scripts to control those activities. However, it does not include the work's System Libraries, or general-purpose tools or generally available free programs which are used unmodified in performing those activities but which are not part of the work. For example, Corresponding Source includes interface definition files associated with source files for the work, and the source code for shared libraries and dynamically linked subprograms that the work is specifically designed to require, such as by intimate data communication or control flow between those subprograms and other parts of the work.

The Corresponding Source need not include anything that users can regenerate automatically from other parts of the Corresponding Source.

The Corresponding Source for a work in source code form is that same work.

2. Basic Permissions.

All rights granted under this License are granted for the term of copyright on the Program, and are irrevocable provided the stated conditions are met. This License explicitly affirms your unlimited permission to run the unmodified Program. The output from running a covered work is covered by this License only if the output, given its content, constitutes a covered work. This License acknowledges your rights of fair use or other equivalent, as provided by copyright law.

You may make, run and propagate covered works that you do not convey, without conditions so long as your license otherwise remains in force. You may convey covered works to others for the sole purpose of having them make modifications exclusively for you, or provide you with facilities for running those works, provided that you comply with the terms of this License in conveying all material for which you do not control copyright. Those thus making or running the covered works for you must do so exclusively on your behalf, under your direction and control, on terms that prohibit them from making any copies of your copyrighted material outside their relationship with you.

Conveying under any other circumstances is permitted solely under the conditions stated below. Sublicensing is not allowed; section 10 makes it unnecessary.

3. Protecting Users' Legal Rights From Anti-Circumvention Law.

No covered work shall be deemed part of an effective technological measure under any applicable law fulfilling obligations under article 11 of the WIPO copyright treaty adopted on 20 December 1996, or similar laws prohibiting or restricting circumvention of such measures.

When you convey a covered work, you waive any legal power to forbid circumvention of technological measures to the extent such circumvention is effected by exercising rights under this License with respect to the covered work, and you disclaim any intention to limit operation or modification of the work as a means of enforcing, against the work's users, your or third parties' legal rights to forbid circumvention of technological measures.

4. Conveying Verbatim Copies.

You may convey verbatim copies of the Program's source code as you receive it, in any medium, provided that you conspicuously and appropriately publish on each copy an appropriate copyright notice; keep intact all notices stating that this License and any non-permissive terms added in accord with section 7 apply to the code; keep intact all notices of the absence of any warranty; and give all recipients a copy of this License along with the Program.

You may charge any price or no price for each copy that you convey, and you may offer support or warranty protection for a fee.

5. Conveying Modified Source Versions.

You may convey a work based on the Program, or the modifications to produce it from the Program, in the form of source code under the terms of section 4, provided that you also meet all of these conditions:

- a) The work must carry prominent notices stating that you modified it, and giving a relevant date.
- b) The work must carry prominent notices stating that it is released under this License and any conditions added under section 7. This requirement modifies the requirement in section 4 to “keep intact all notices”.

- c) You must license the entire work, as a whole, under this License to anyone who comes into possession of a copy. This License will therefore apply, along with any applicable section 7 additional terms, to the whole of the work, and all its parts, regardless of how they are packaged. This License gives no permission to license the work in any other way, but it does not invalidate such permission if you have separately received it.
- d) If the work has interactive user interfaces, each must display Appropriate Legal Notices; however, if the Program has interactive interfaces that do not display Appropriate Legal Notices, your work need not make them do so.

A compilation of a covered work with other separate and independent works, which are not by their nature extensions of the covered work, and which are not combined with it such as to form a larger program, in or on a volume of a storage or distribution medium, is called an “aggregate” if the compilation and its resulting copyright are not used to limit the access or legal rights of the compilation's users beyond what the individual works permit. Inclusion of a covered work in an aggregate does not cause this License to apply to the other parts of the aggregate.

6. Conveying Non-Source Forms.

You may convey a covered work in object code form under the terms of sections 4 and 5, provided that you also convey the machine-readable Corresponding Source under the terms of this License, in one of these ways:

- a) Convey the object code in, or embodied in, a physical product (including a physical distribution medium), accompanied by the Corresponding Source fixed on a durable physical medium customarily used for software interchange.
- b) Convey the object code in, or embodied in, a physical product (including a physical distribution medium), accompanied by a written offer, valid for at least three years and valid for as long as you offer spare parts or customer support for that product model, to give anyone who possesses the object code either (1) a copy of the Corresponding Source for all the software in the product that is covered by this License, on a durable physical medium customarily used for software interchange, for a price no more than your reasonable cost of physically performing this conveying of source, or (2) access to copy the Corresponding Source from a network server at no charge.
- c) Convey individual copies of the object code with a copy of the written offer to provide the Corresponding Source. This alternative is allowed only occasionally and noncommercially, and only if you received the object code with such an offer, in accord with subsection 6b.
- d) Convey the object code by offering access from a designated place (gratis or for a charge), and offer equivalent access to the Corresponding Source in the same way through the same place at no further charge. You need not require recipients to copy the Corresponding Source along with the object code. If the place to copy the object code is a network server, the Corresponding Source may be on a different server (operated by you or a third party) that supports equivalent copying facilities, provided you maintain clear directions next to the object code saying where to find the Corresponding Source. Regardless of what server hosts the

Corresponding Source, you remain obligated to ensure that it is available for as long as needed to satisfy these requirements.

- e) Convey the object code using peer-to-peer transmission, provided you inform other peers where the object code and Corresponding Source of the work are being offered to the general public at no charge under subsection 6d.

A separable portion of the object code, whose source code is excluded from the Corresponding Source as a System Library, need not be included in conveying the object code work.

A “User Product” is either (1) a “consumer product”, which means any tangible personal property which is normally used for personal, family, or household purposes, or (2) anything designed or sold for incorporation into a dwelling. In determining whether a product is a consumer product, doubtful cases shall be resolved in favor of coverage. For a particular product received by a particular user, “normally used” refers to a typical or common use of that class of product, regardless of the status of the particular user or of the way in which the particular user actually uses, or expects or is expected to use, the product. A product is a consumer product regardless of whether the product has substantial commercial, industrial or non-consumer uses, unless such uses represent the only significant mode of use of the product.

“Installation Information” for a User Product means any methods, procedures, authorization keys, or other information required to install and execute modified versions of a covered work in that User Product from a modified version of its Corresponding Source. The information must suffice to ensure that the continued functioning of the modified object code is in no case prevented or interfered with solely because modification has been made.

If you convey an object code work under this section in, or with, or specifically for use in, a User Product, and the conveying occurs as part of a transaction in which the right of possession and use of the User Product is transferred to the recipient in perpetuity or for a fixed term (regardless of how the transaction is characterized), the Corresponding Source conveyed under this section must be accompanied by the Installation Information. But this requirement does not apply if neither you nor any third party retains the ability to install modified object code on the User Product (for example, the work has been installed in ROM).

The requirement to provide Installation Information does not include a requirement to continue to provide support service, warranty, or updates for a work that has been modified or installed by the recipient, or for the User Product in which it has been modified or installed. Access to a network may be denied when the modification itself materially and adversely affects the operation of the network or violates the rules and protocols for communication across the network.

Corresponding Source conveyed, and Installation Information provided, in accord with this section must be in a format that is publicly documented (and with an implementation available to the public in source code form), and must require no special password or key for unpacking, reading or copying.

7. Additional Terms.

“Additional permissions” are terms that supplement the terms of this License by making exceptions from one or more of its conditions. Additional permissions that are applicable to the entire Program shall be treated as though they were included in this License, to the extent that they are valid under applicable law. If additional permissions apply only to part of the Program, that part may be used separately under those permissions, but the entire Program remains governed by this License without regard to the additional permissions.

When you convey a copy of a covered work, you may at your option remove any additional permissions from that copy, or from any part of it. (Additional permissions may be written to require their own removal in certain cases when you modify the work.) You may place additional permissions on material, added by you to a covered work, for which you have or can give appropriate copyright permission.

Notwithstanding any other provision of this License, for material you add to a covered work, you may (if authorized by the copyright holders of that material) supplement the terms of this License with terms:

- a) Disclaiming warranty or limiting liability differently from the terms of sections 15 and 16 of this License; or
- b) Requiring preservation of specified reasonable legal notices or author attributions in that material or in the Appropriate Legal Notices displayed by works containing it; or
- c) Prohibiting misrepresentation of the origin of that material, or requiring that modified versions of such material be marked in reasonable ways as different from the original version; or
- d) Limiting the use for publicity purposes of names of licensors or authors of the material; or
- e) Declining to grant rights under trademark law for use of some trade names, trademarks, or service marks; or
- f) Requiring indemnification of licensors and authors of that material by anyone who conveys the material (or modified versions of it) with contractual assumptions of liability to the recipient, for any liability that these contractual assumptions directly impose on those licensors and authors.

All other non-permissive additional terms are considered “further restrictions” within the meaning of section 10. If the Program as you received it, or any part of it, contains a notice stating that it is governed by this License along with a term that is a further restriction, you may remove that term. If a license document contains a further restriction but permits relicensing or conveying under this License, you may add to a covered work material governed by the terms of that license document, provided that the further restriction does not survive such relicensing or conveying.

If you add terms to a covered work in accord with this section, you must place, in the relevant source files, a statement of the additional terms that apply to those files, or a notice indicating where to find the applicable terms.

Additional terms, permissive or non-permissive, may be stated in the form of a separately written license, or stated as exceptions; the above requirements apply either way.

8. Termination.

You may not propagate or modify a covered work except as expressly provided under this License. Any attempt otherwise to propagate or modify it is void, and will automatically terminate your rights under this License (including any patent licenses granted under the third paragraph of section 11).

However, if you cease all violation of this License, then your license from a particular copyright holder is reinstated (a) provisionally, unless and until the copyright holder explicitly and finally terminates your license, and (b) permanently, if the copyright holder fails to notify you of the violation by some reasonable means prior to 60 days after the cessation.

Moreover, your license from a particular copyright holder is reinstated permanently if the copyright holder notifies you of the violation by some reasonable means, this is the first time you have received notice of violation of this License (for any work) from that copyright holder, and you cure the violation prior to 30 days after your receipt of the notice.

Termination of your rights under this section does not terminate the licenses of parties who have received copies or rights from you under this License. If your rights have been terminated and not permanently reinstated, you do not qualify to receive new licenses for the same material under section 10.

9. Acceptance Not Required for Having Copies.

You are not required to accept this License in order to receive or run a copy of the Program. Ancillary propagation of a covered work occurring solely as a consequence of using peer-to-peer transmission to receive a copy likewise does not require acceptance. However, nothing other than this License grants you permission to propagate or modify any covered work. These actions infringe copyright if you do not accept this License. Therefore, by modifying or propagating a covered work, you indicate your acceptance of this License to do so.

10. Automatic Licensing of Downstream Recipients.

Each time you convey a covered work, the recipient automatically receives a license from the original licensors, to run, modify and propagate that work, subject to this License. You are not responsible for enforcing compliance by third parties with this License.

An “entity transaction” is a transaction transferring control of an organization, or substantially all assets of one, or subdividing an organization, or merging organizations. If propagation of a covered work results from an entity transaction, each party to that transaction who receives a copy of the work also receives whatever licenses to the work the party's predecessor in interest had or could give under the previous paragraph, plus a right to possession of the Corresponding Source of the work from the predecessor in interest, if the predecessor has it or can get it with reasonable efforts.

You may not impose any further restrictions on the exercise of the rights granted or affirmed under this License. For example, you may not impose a license fee, royalty, or other charge for exercise of rights granted under this License, and you may not initiate litigation (including a cross-claim or counterclaim in a lawsuit) alleging that any patent claim is infringed by making, using, selling, offering for sale, or importing the Program or any portion of it.

11. Patents.

A “contributor” is a copyright holder who authorizes use under this License of the Program or a work on which the Program is based. The work thus licensed is called the contributor's “contributor version”.

A contributor's “essential patent claims” are all patent claims owned or controlled by the contributor, whether already acquired or hereafter acquired, that would be infringed by some manner, permitted by this License, of making, using, or selling its contributor version, but do not include claims that would be infringed only as a consequence of further modification of the contributor version. For purposes of this definition, “control” includes the right to grant patent sublicenses in a manner consistent with the requirements of this License.

Each contributor grants you a non-exclusive, worldwide, royalty-free patent license under the contributor's essential patent claims, to make, use, sell, offer for sale, import and otherwise run, modify and propagate the contents of its contributor version.

In the following three paragraphs, a “patent license” is any express agreement or commitment, however denominated, not to enforce a patent (such as an express permission to practice a patent or covenant not to sue for patent infringement). To “grant” such a patent license to a party means to make such an agreement or commitment not to enforce a patent against the party.

If you convey a covered work, knowingly relying on a patent license, and the Corresponding Source of the work is not available for anyone to copy, free of charge and under the terms of this License, through a publicly available network server or other readily accessible means, then you must either (1) cause the Corresponding Source to be so available, or (2) arrange to deprive yourself of the benefit of the patent license for this particular work, or (3) arrange, in a manner consistent with the requirements of this License, to extend the patent license to downstream recipients. “Knowingly relying” means you have actual knowledge that, but for the patent license, your conveying the covered work in a country, or your recipient's use of the covered work in a country, would infringe one or more identifiable patents in that country that you have reason to believe are valid.

If, pursuant to or in connection with a single transaction or arrangement, you convey, or propagate by procuring conveyance of, a covered work, and grant a patent license to some of the parties receiving the covered work authorizing them to use, propagate, modify or convey a specific copy of the covered work, then the patent license you grant is automatically extended to all recipients of the covered work and works based on it.

A patent license is “discriminatory” if it does not include within the scope of its coverage, prohibits the exercise of, or is conditioned on the non-exercise of one or more of the rights that are specifically granted under this License. You may not convey a covered work if you are a party to an arrangement with a third party that is in the business of distributing software, under which you make payment to the third party based on the extent of your activity of conveying the work, and under which the third party grants, to any of the parties who would receive the covered work from you, a discriminatory patent license (a) in connection with copies of the covered work conveyed by you (or copies made from those copies), or (b) primarily for and in connection with specific products or compilations that contain the covered work, unless you entered into that arrangement, or that patent license was granted, prior to 28 March 2007.

Nothing in this License shall be construed as excluding or limiting any implied license or other defenses to infringement that may otherwise be available to you under applicable patent law.

12. No Surrender of Others' Freedom.

If conditions are imposed on you (whether by court order, agreement or otherwise) that contradict the conditions of this License, they do not excuse you from the conditions of this License. If you cannot convey a covered work so as to satisfy simultaneously your obligations under this License and any other pertinent obligations, then as a consequence you may not convey it at all. For example, if you agree to terms that obligate you to collect a royalty for further conveying from those to whom you convey the Program, the only way you could satisfy both those terms and this License would be to refrain entirely from conveying the Program.

13. Use with the GNU Affero General Public License.

Notwithstanding any other provision of this License, you have permission to link or combine any covered work with a work licensed under version 3 of the GNU Affero General Public License into a single combined work, and to convey the resulting work. The terms of this License will continue to apply to the part which is the covered work, but the special requirements of the GNU Affero General Public License, section 13, concerning interaction through a network will apply to the combination as such.

14. Revised Versions of this License.

The Free Software Foundation may publish revised and/or new versions of the GNU General Public License from time to time. Such new versions will be similar in spirit to the present version, but may differ in detail to address new problems or concerns.

Each version is given a distinguishing version number. If the Program specifies that a certain numbered version of the GNU General Public License “or any later version” applies to it, you have the option of following the terms and conditions either of that numbered version or of any later version published by the Free Software Foundation. If the Program does not specify a version number of the GNU General Public License, you may choose any version ever published by the Free Software Foundation.

If the Program specifies that a proxy can decide which future versions of the GNU General Public License can be used, that proxy's public statement of acceptance of a version permanently authorizes you to choose that version for the Program.

Later license versions may give you additional or different permissions. However, no additional obligations are imposed on any author or copyright holder as a result of your choosing to follow a later version.

15. Disclaimer of Warranty.

THERE IS NO WARRANTY FOR THE PROGRAM, TO THE EXTENT PERMITTED BY APPLICABLE LAW. EXCEPT WHEN OTHERWISE STATED IN WRITING THE COPYRIGHT HOLDERS AND/OR OTHER PARTIES PROVIDE THE PROGRAM “AS IS” WITHOUT WARRANTY OF ANY KIND, EITHER EXPRESSED OR IMPLIED, INCLUDING, BUT NOT LIMITED TO, THE IMPLIED WARRANTIES OF MERCHANTABILITY AND FITNESS FOR A PARTICULAR PURPOSE. THE ENTIRE RISK AS TO THE QUALITY AND PERFORMANCE OF THE PROGRAM IS WITH YOU. SHOULD THE PROGRAM PROVE DEFECTIVE, YOU ASSUME THE COST OF ALL NECESSARY SERVICING, REPAIR OR CORRECTION.

16. Limitation of Liability.

IN NO EVENT UNLESS REQUIRED BY APPLICABLE LAW OR AGREED TO IN WRITING WILL ANY COPYRIGHT HOLDER, OR ANY OTHER PARTY WHO MODIFIES AND/OR CONVEYS THE PROGRAM AS PERMITTED ABOVE, BE LIABLE TO YOU FOR DAMAGES, INCLUDING ANY GENERAL, SPECIAL, INCIDENTAL OR CONSEQUENTIAL DAMAGES ARISING OUT OF THE USE OR INABILITY TO USE THE PROGRAM (INCLUDING BUT NOT LIMITED TO LOSS OF DATA OR DATA BEING RENDERED INACCURATE OR LOSSES SUSTAINED BY YOU OR THIRD PARTIES OR A FAILURE OF THE PROGRAM TO OPERATE WITH ANY OTHER PROGRAMS), EVEN IF SUCH HOLDER OR OTHER PARTY HAS BEEN ADVISED OF THE POSSIBILITY OF SUCH DAMAGES.

17. Interpretation of Sections 15 and 16.

If the disclaimer of warranty and limitation of liability provided above cannot be given local legal effect according to their terms, reviewing courts shall apply local law that most closely approximates an absolute waiver of all civil liability in connection with the Program, unless a warranty or assumption of liability accompanies a copy of the Program in return for a fee.

END OF TERMS AND CONDITIONS
